# Procedural Support for Neurodivergent Children During Medical Procedures: A Scoping Review

**DOI:** 10.1007/s10567-026-00562-w

**Published:** 2026-02-24

**Authors:** Mari Takashima, Harriet Robertson, Karin Plummer, Catherine Kotzur, Adel Wesley, Alexandra Donaldson, Henrik Hjelmgren, Anna Stålberg, Lisa Burgess, Alexander J. Friedman, Laurel Mimmo, Jade Choi, Natalie Barker, Sherelyn Gooley, Nicki Walsh, Vanessa E. Cobham, Amanda Ullman

**Affiliations:** 1 School of Nursing, Midwifery and Social Work, Faculty of Health and Behavioural Sciences, The University of Queensland, St Lucia, QLD Australia; 2 Queensland Children’s Hospital, Children’s Health Queensland Hospital and Health Service, South Brisbane, QLD Australia; 3 Child Health Research Centre, Faculty of Medicine, The University of Queensland, 62 Graham Street, South Brisbane, QLD 4101 Australia; 4 Griffith Biostatistical Unit, Griffith University, Brisbane, QLD Australia; 5 School of Nursing and Midwifery, Griffith University, Brisbane, QLD Australia; 6 Astrid Lindgren’s Children’s Hospital, Karolinska University Hospital, Stockholm, Sweden; 7 Paediatric Healthcare Science, Childhood Cancer Research Unit, Department of Women’s and Children’s Health, Karolinska Institute, Stockholm, Sweden; 8 Boston Medical Center, Boston, MA US; 9 Nursing Research Unit, Sydney Children’s Hospitals Network, Sydney, NSW Australia; 10 Centre for Health Systems and Safety Research, Australian Institute of Health Innovation, Macquarie University, Sydney, NSW Australia; 11 Sydney Medical School, Faculty of Medicine and Health, University of Sydney, Sydney, NSW Australia; 12 Herston Health Sciences Library, The University of Queensland, Brisbane, QLD 4072 Australia; 13 School of Psychology, Faculty of Health, Medicine and Behavioural Sciences, The University of Queensland, South Brisbane, Australia; 14 Children’s Health Queensland Child and Youth Mental Health Service, South Brisbane, Australia

**Keywords:** Neurodivergent, Procedural distress, Paediatric, Healthcare access, Pain and distress management, Procedural

## Abstract

Neurodivergent children face unique challenges during medical procedures due to distinct sensory processing patterns and communication difficulties. Evidence-based interventions for procedural pain/distress may inadequately address their specific needs, leading to undertreated distress and negative healthcare experiences.

Following Joanna Briggs Institute methodology, we conducted comprehensive searches across six databases on January 10, 2025, for studies published 2014–2025 focusing on neurodivergent children (0–21 years) undergoing medical procedures. Two independent reviewers screened studies and extracted data, with results presented as a narrative synthesis with evidence mapping.

From 14,393 initial records, 144 studies met the inclusion criteria. Most studies (n = 121, 84.0%) focused on autism spectrum disorder, with limited representation of other neurodivergent diagnoses. Hospital outpatient settings (n = 93, 64.6%) and dental specialty (n = 50, 34.7%) were most commonly studied. The most frequently used support strategies were visit preparation and support (n = 61, 42.4%), pharmacological agents (n = 48, 33.3%), and patient care plans/pathways (n = 43, 29.9%). Few studies used validated pain and distress assessment tools, with only 4.2% (n = 6) reporting child-reported pain measures. While 74.3% (n = 107) of studies reported distress outcomes, these were primarily observational rather than validated measures.

Findings highlight significant gaps in procedural support strategies for neurodivergent children, particularly for conditions beyond autism. There is a critical need for research using validated pain and distress measures, especially those capturing the child’s perspective. Future studies should prioritise diverse neurodivergent populations, incorporate structured assessment tools, and evaluate tailored interventions across wider clinical settings.

**Supplementary Information:**

The online version contains supplementary material available at 10.1007/s10567-026-00562-w.

## Introduction

Children frequently experience pain and distress during medical procedures, especially those involving needles. While evidence-based interventions exist to minimise procedural distress, it remains under-managed (Birnie et al., 2014), leading to anxiety and trauma responses (Lerwick, 2016). Procedural distress is a multifactorial unpleasant emotional experience that encompasses any type of negative effect (including anxiety, fear, stress, and pain) associated with medical procedures (Birnie et al., 2018; McGrath et al., 2008). The relationships between these factors are complex, with distress emerging from interactions between pain perception, anxiety, fear, and stress responses before, during, and after procedures. Typically, this distress manifests as anticipatory and situational reactions, characterised by tension, apprehension, and heightened physiological arousal. These responses can influence not only the immediate experience but also future healthcare interactions and long-term outcomes (Eccleston et al., 2021; Leblanc et al., 2024). Childhood procedural distress typically develops through a combination of anticipatory fear, negative past experiences, and developmental vulnerabilities. When left unaddressed, this distress can lead to increased pain perception, decreased cooperation with medical care, and lasting traumatic responses (Lerwick, 2016). The impacts extend beyond the immediate procedure, influencing both future healthcare experiences and long-term health outcomes (Eccleston et al., 2021). Up to 25% of adults have a fear of needles, developed primarily in childhood, with about 10% avoiding healthcare due to this fear (McMurtry et al., 2016; Taddio, 2019).

Children with neurodivergent diagnoses, as classified by the DSM-5-TR (American Psychiatric Association, 2022), including autism spectrum disorder (ASD), attention deficit hyperactivity disorder (ADHD), intellectual disabilities, communication disorders, and specific learning disorders, face particular challenges during medical procedures (da Silva et al., 2024; Ely et al., 2016; Johnson et al., 2023; Leblanc et al., 2024; Trottier et al., 2022). These conditions affect approximately 16.7% of children in high-income countries and 7.5 per 1000 children in low and middle-income countries, representing a significant portion of the paediatric population worldwide (Bitta et al., 2017; Li et al., 2023). Their unique sensory processing patterns, communication difficulties, and behavioural responses can make managing procedural distress more complex (Birnie et al., 2018; Cheng et al., 2018; Doyle et al., 2022; Harris et al., 2024). Evidence-based interventions for procedural support typically fall into three main categories: pharmacological (such as topical anaesthesia and sedation), physical (such as positioning, heat/cold applications, and mechanical devices), and psychological (such as distraction, preparation, and hypnosis) (Birnie et al., 2018; Friedrichsdorf & Goubert, 2020). While these interventions have demonstrated effectiveness in reducing procedural distress for neurotypical children (Birnie et al., 2018), there is limited evidence evaluating their efficacy and implementation specifically for neurodivergent populations, with existing studies showing significant gaps in comprehensive evaluation across healthcare settings (Harris et al., 2024). Additionally, pain assessment and communication methods for neurodivergent children are frequently inadequate or entirely absent in clinical practice, further complicating effective procedural support for this vulnerable population (Barney et al., 2020).

A search of PubMed, the Cochrane Database of Systematic Reviews, and JBI Evidence Synthesis revealed that while reviews have examined procedural support strategies specifically for autistic children (Harris et al., 2024; Johnson et al., 2023; Leblanc et al., 2024), no comprehensive reviews have examined procedural support across the full spectrum of neurodivergent diagnoses as defined by DSM-5-TR (American Psychiatric Association, 2022). This gap is particularly concerning given that neurodivergent conditions collectively affect approximately 16.7% of children in high-income countries (Li et al., 2023), encompassing not only autism but also ADHD, communication disorders, intellectual disabilities, specific learning disorders, and motor disorders. Understanding whether research evidence exists equitably across all neurodivergent populations, or whether certain diagnoses are systematically understudied, is essential for identifying research priorities and ensuring that all neurodivergent children receive evidence-based procedural support. A comprehensive scoping review across all neurodivergent conditions can reveal such disparities in the evidence base and guide future research efforts toward underrepresented populations.

## Review Question

What is the evidence for procedural support interventions for managing pain/distress in neurodivergent children and adolescents (0–21 years) undergoing medical procedures, what methods are used to assess procedural pain/distress in this population, and what are the identified barriers and facilitators to implementing effective procedural support for this group?

## Methods

The methodological design of this scoping review protocol is congruent with the Joanna Briggs Institute Manual for Evidence Synthesis and Scoping Review Framework (Aromataris & Munn, 2020), and reported in accordance with Preferred Reporting Items for Systematic Reviews and Meta-analysis protocols specific to scoping reviews (PRISMA-ScR) (Liberati et al., 2009).

### Search Strategy

The search strategy was developed in two phases. A preliminary search of PubMed and CINAHL was conducted to determine essential search terms and keywords for inclusion. This initial search helped identify relevant index terms/subject headings, and commonly used terminology in the field of procedural support for neurodivergent children. A second systematic search was completed across PubMed, Embase, PsycINFO, Cochrane Central Register of Controlled Trials (CENTRAL), Web of Science, and CINAHL using all the identified keywords, synonyms, and subject indexing terms, (Supplementary Material 1). The published results of eligible studies (see Table 1 for eligibility criteria) were sourced and imported into Covidence Systematic Review Software (version 2.0, Veritas Health Innovation), for full-text screening by the same two independent reviewers. We used DSM-5-TR (American Psychiatric Association, 2022) categories to define neurodivergent conditions, including conditions associated with prenatal substance exposure when classified within these diagnostic categories by original study authors. Diagnostic categories were accepted as defined by the original authors during screening, then harmonised to DSM-5-TR terminology during analysis. Regional terminology variations (e.g., UK ‘learning disorders’ referring to intellectual disabilities) were reconciled through consultation with a clinical psychologist. The 0–21 year age range aligned with the American Academy of Pediatrics’ definition of the paediatric population. Studies with mixed populations were included if neurodivergent data were reported separately or if ≥ 75% of participants fell within our age range. The 2014–2025 timeframe captured contemporary practice relevant to current clinical care. Studies examining pain assessment methods and healthcare experiences were included when they provided essential context for evaluating procedural support effectiveness and identifying implementation barriers. Secondary research studies were excluded from data extraction to avoid duplication; however, their reference lists were hand-searched to identify additional primary studies. Reference lists of all included studies were screened for additional eligible studies, which subsequently underwent the same assessment process for inclusion eligibility.

**Table 1:** Eligibility criteria

Category	Inclusion criteria	Exclusion criteria
Participants	Children aged 0–21 years; or/and parents/caregivers Diagnosed with neurodivergent conditions, including: Autism Spectrum Disorder (ASD) Attention Deficit Hyperactivity Disorder (ADHD) Attention Deficit Disorder (ADD) Communication disorders Intellectual Disorders Neurodevelopmental motor disorders (e.g., cerebral palsy, Down’s syndrome) Specific Learning Disorders (e.g., dyslexia)	Studies focusing solely on neurotypical children
Concept	Procedural support strategies Pain and distress assessment methods Healthcare experiences of children and family Outcomes related to procedural support, including pain, distress, global judgement of satisfaction, and adverse events	Studies that do not include procedural support, pain/distress management, or healthcare experiences
Context	Any healthcare setting (hospitals, clinics, community health centres) Any geographical location Any type of medical procedure Studies published between 2014–2025 English language	Studies published before 2014 Non-English language studies

### Types of Sources

This scoping review considered a comprehensive range of study designs, including experimental studies (randomised controlled trials, non-randomised controlled trials, quasi-experimental studies, before and after studies, and interrupted time-series studies), observational studies (prospective and retrospective cohort studies, case–control studies, cross-sectional studies, case series, and case reports), qualitative research (phenomenology, grounded theory, ethnography, action research, and qualitative description), mixed-methods studies, and secondary research (systematic reviews, meta-analyses, scoping reviews, and integrative reviews). Only peer-reviewed publications were included; unpublished theses, non-peer-reviewed publications, and opinion pieces were excluded. All included sources had to be available in full text or as a structured abstract with sufficient methodological detail to enable data extraction.

### Study Selection

Records retrieved from the database searches were imported into EndNote V.20.2.0.17373 for initial management. These records were then transferred into Covidence Systematic Review Software (Veritas Health Innovation) for duplicate removal and screening processes. Two independent authors conducted the title and abstract screening, with arbitration by a third author in cases of disagreement. Full-text publications were obtained for all potentially eligible studies and reviewed by the same two independent reviewers. The results of the search and the study inclusion process were reported in full in the final scoping review and presented in a Preferred Reporting Items for Systematic Reviews and Meta-analyses extension for scoping review (PRISMA-ScR) flow diagram(Liberati et al., 2009).

### Data Extraction

Data were extracted from included papers by two or more independent reviewers using a data extraction tool developed specifically for this review (Supplementary Table 1). The data extraction tool was tested by two independent authors for two studies to ensure robustness, prior to full data extraction completion. Authors were contacted for additional information when required. The tool captured detailed information about study characteristics, participant demographics (including author-defined neurodivergent diagnoses), procedural settings, support strategies, assessment methods, outcomes, and importantly, author-defined barriers and facilitators to implementing effective procedural support.

### Data Analysis and Presentation

Extracted data were analysed using descriptive statistics (counts, percentages, medians, ranges, IQRs) relevant to the data characteristics and distribution. For qualitative data, including author-defined barriers and facilitators, a content analysis approach was employed to identify, code, and categorise patterns within the text (Hsieh & Shannon, 2005). Results were presented in tabular format for study demographics and key findings, accompanied by a descriptive summary demonstrating how the results pertained to the scoping review objectives. The figures were created using RAWGraphs (version 2.0, Density Design, 2021) and Tableau (version 2024.3, Salesforce, 2024), while Airtable (Airtable Inc., San Francisco, CA, USA) was used for data cleaning and organisation. Stata (version 18, StataCorp, 2024) was used for quantitative data analysis.

## Results

### Study Selection

**Fig. 1:**
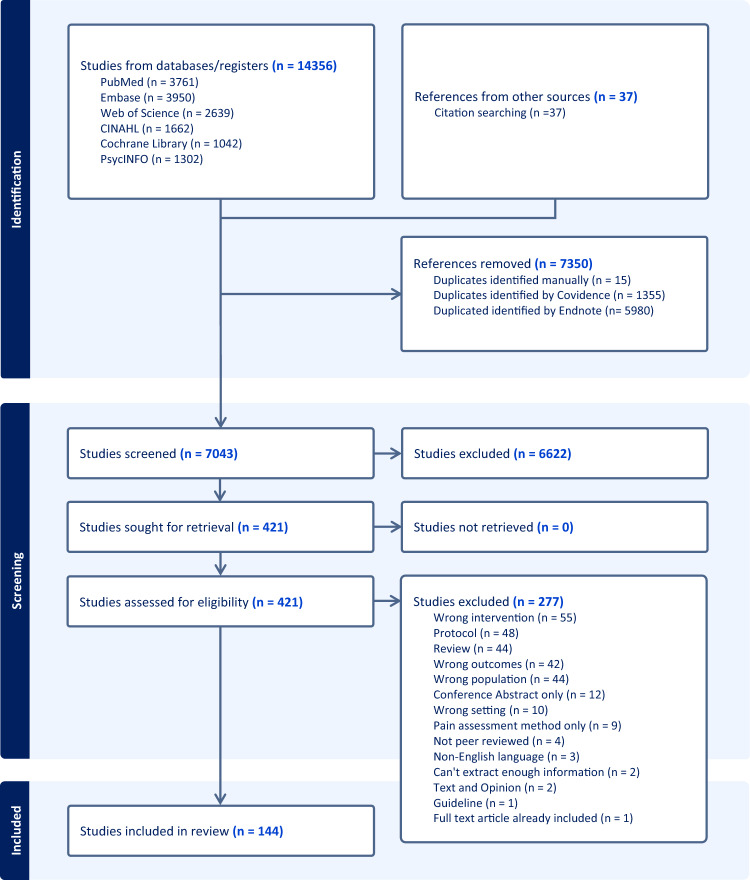
PRISMA flowchart of included studies

A comprehensive search of six databases and additional sources yielded a total of 14,393 records (14,356 from databases and 37 from other sources). After removing 7,350 duplicates (15 identified manually, 1,355 identified by Covidence, and 5,980 identified by EndNote), 7,043 studies were screened for eligibility. Of these, 6,622 were excluded at the title and abstract screening phase. The remaining 421 studies were sought for full-text retrieval, all of which were successfully obtained. Upon full-text assessment, 277 studies were excluded due to: wrong intervention (n = 55), protocol papers (n = 48), review (n = 44), wrong population (n = 44), wrong outcomes (n = 42), conference abstracts only (n = 12), wrong setting (n = 10), pain assessment method only (n = 9), not peer reviewed (n = 4), non-English language (n = 3), text and opinion (n = 2), insufficient information for data extraction (n = 2), guideline (n = 1), and full-text article already included (n = 1). Finally, 144 studies met the inclusion criteria and were included in this scoping review (Fig. 1; Supplementary Materials 2 and 3).

### Study Characteristics

**Fig. 2:**
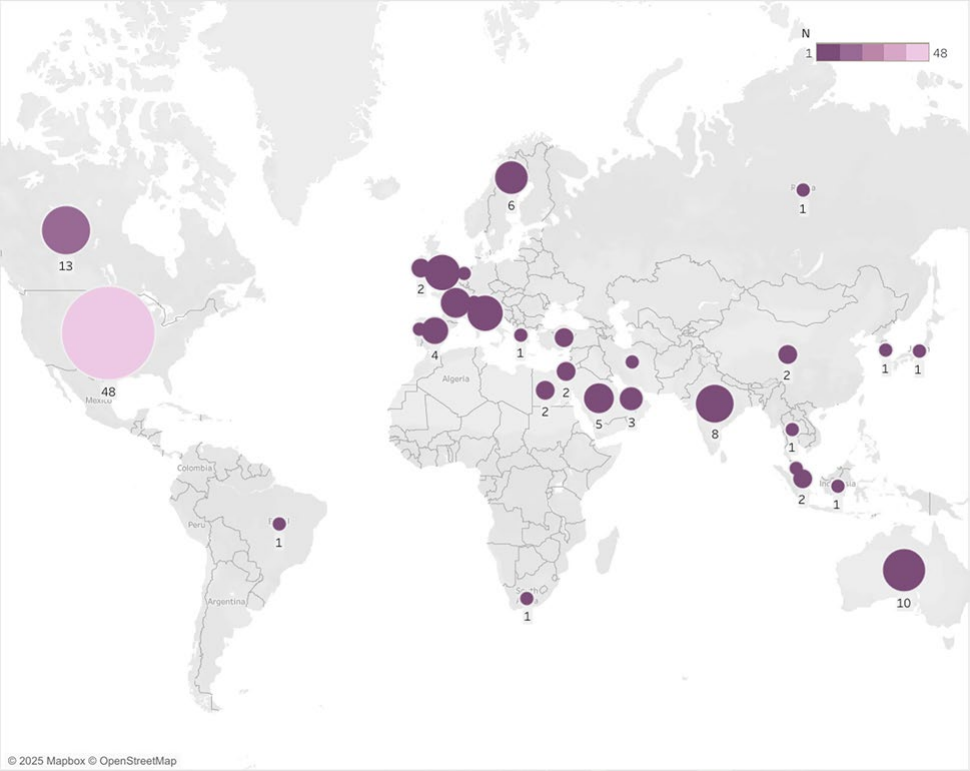
Map of countries in which the study was conducted (N = 144)*,*2 studies had multiple countries

The included studies were published between 2014 and 2025, with the majority published in 2023 (n = 18, 12.5%) and 2024 (n = 22, 15.3%), indicating increasing research interest in recent years (Table 1). The geographical distribution of studies on procedural support (Fig. 2) for neurodivergent children shows a substantial imbalance, with the United States contributing the largest proportion (n = 48, 33.3%) of all publications. Other English-speaking countries, including Canada (n = 13, 9.0%), Australia (n = 10, 6.9%), and the UK (n = 7, 4.9%) were also major contributors. European representation was noteworthy through Italy and UK (n = 7, 4.9% each), Sweden (n = 6, 4.2%), and France (n = 5, 3.5%), while Middle Eastern contributions came primarily from Saudi Arabia (n = 5, 3.5%). The significant underrepresentation of research from South America, Africa, and most of Asia highlights gaps in our understanding of culturally diverse approaches to supporting neurodivergent children during medical procedures (Table 2).

**Table 2:** Table of study characteristics (N = 144)

Characteristics		N (%)
Year of publication	2014	11 (7.6)
	2015	6 (4.2)
	2016	13 (9.0)
	2017	16 (11.1)
	2018	11 (7.6)
	2019	15 (10.4)
	2020	14 (9.7)
	2021	9 (6.3)
	2022	9 (6.3)
	2023	18 (12.5)
	2024	22 (15.3)
Study Design	Qualitative research	26 (18.1)
	Retrospective Cohort study	21 (14.6)
	Randomised controlled trial	20 (13.9)
	Case study/series	17 (11.8)
	Prospective Cohort study	13 (9.0)
	Mixed Methods	12 (8.3)
	Non-randomised experimental study	8 (5.6)
	Quality Initiative	8 (5.6)
	Survey	7 (4.9)
	Cross sectional study	5 (3.5)
	Cross-over study	4 (2.8)
	Case Control Study	1 (0.7)
	Modified Delphi method	1 (0.7)
	Randomised cross-over trial	1 (0.7)
Setting*	Hospital (outpatient)	93 (64.6)
	Hospital (inpatient)	41 (28.5)
	Out of hospital (community)	21 (14.6)
Clinical Speciality*	Dental	50 (34.7)
	Medical/Surgical	46 (31.9)
	Perioperative	17 (11.8)
	Radiology	14 (9.7)
	Emergency Department	10 (6.9)
	Neurology	5 (3.5)
	Psychiatry	4 (2.8)
	Rehabilitation	3 (2.1)
	Anaesthesiology	2 (1.4)
Funding*	Not stated	69 (47.9)
	Government	26 (18.6)
	None	22 (15.3)
	Hospital	14 (9.7)
	University	12 (8.3)
	Philanthropy	12 (8.3)
Number of children reported		11,484
Number of parents reported		1,690
Number of clinicians reported		839
Age group of children*	Baby (0–2 years old)	23 (16.0)
	Toddler (18 months -3 years)	47 (32.6)
	Pre-school (4–5 years)	81 (56.3)
	School-aged (6–12 years)	119 (82.6)
	Adolescent (13 yeas and older)	89 (61.8)
Neurodiversity diagnosis* (DSM-5-TR)	Autism Spectrum Disorder	121 (84.0)
	Intellectual Disorders (including Down’s Syndrome)	41 (28.5)
	Attention Deficit Hyperactivity Disorder, including ADD	30 (20.8)
	Neurodevelopmental motor disorders (includes cerebral palsy)	13 (9.0)
	Communication disorders	5 (3.5)
	Specific Learning Disorders (such as dyslexia)	1 (0.7)
	Other^	1 (0.7)
Communication level*	Not Stated	97 (67.4)
	Verbal	35 (24.3)
	Non-verbal	35 (24.3)
	Assisted Communication	13 (9.0)

*ADD* Attention Deficit Disorder

*Can have multiple options; ^reported neurodivergent diagnoses as a single combined category without breaking down specific diagnoses

The most common study designs were qualitative research (n = 26, 18.1%), retrospective cohort studies (n = 21, 14.6%), and randomised controlled trials (n = 20, 13.9%). Most studies were conducted in hospital outpatient settings (n = 93, 64.6%), with hospital inpatient settings accounting for n = 41 (28.5%) of studies. Dental procedures represented the largest clinical specialty group (n = 50, 34.7%), followed by medical/surgical procedures (n = 46, 31.9%), perioperative care (n = 17, 11.8%), and radiology (n = 14, 9.7%). Nearly half of the studies (n = 69, 47.9%) did not state their funding source, while government funding was reported in 18.6% (n = 26) of studies.

A total of 11,484 children were reported across the included studies, alongside 1,690 parents and 839 clinicians. Age-related data were available for a subset of the children and were categorised into five developmental groups. Most were school-aged children (6–12 years; n = 119; 82.6%) and adolescents (13 years or older; n = 89; 61.8%). The majority of studies (n = 121, 84.0%) focused on children with Autism Spectrum Disorder (ASD), with significantly less representation of other neurodivergent conditions: intellectual disorders including Down’s Syndrome (n = 41, 28.5%), Attention Deficit Hyperactivity Disorders including ADD (n = 30, 20.8%), neurodevelopmental motor disorders including cerebral palsy (n = 13, 9.0%), communication disorders (n = 5, 3.5%), specific learning disorders such as dyslexia (n = 1, 0.7%), and other conditions (n = 1, 0.7%). All diagnostic categories were as defined by the original study authors.

Procedures were most commonly conducted in dental clinics (n = 48, 33.3%), procedure rooms (n = 29, 20.1%), radiology departments (n = 17, 11.8%), operating theatres (n = 14, 9.7%), and emergency departments (n = 10, 6.9%; Table 3). Dental procedures were the most common procedure (n = 61, 42.4%), followed by radiological/diagnostic procedures (n = 40, 27.8%), venipuncture/IV/IM (n = 22, 15.3%), general hospital experience (n = 21, 14.6%), and emergency procedures (n = 8, 5.6%). The duration of procedures varied, with 6 studies (4.2%) lasting less than 15 min, 12 (8.3%) between 15–30 min, 13 (9.0%) between 30–60 min, 8 (5.6%) between 60–120 min, and 13 (9.0%) involving procedures longer than 120 min or multiple sessions. However, the duration was not reported or unclear in most studies (n = 97, 67.4%).

**Table 3:** Table of medical procedures and procedural supports (N = 144)

Characteristics		N (%)
Location of medical procedures*	Dental Clinic	48 (33.3)
	Procedure Room	29 (20.1)
	Outpatient clinic/ Community clinic	18 (12.5)
	Radiology (i.e. MRI)	17 (11.8)
	Operating Theatre	14 (9.7)
	Emergency Department	10 (6.9)
	Multiple Hospital Settings/Wards	14 (9.7)
	Bedside	3 (2.1)
Type of medical procedures*	Dental	61 (42.4)
	Radiological/Diagnostic	40 (27.8)
	Needle-related procedures	22 (15.3)
	General Hospital Experience	21 (14.6)
	Pre-surgery	17 (11.8)
	Emergency	8 (5.6)
	Dressing Changes	2 (1.4)
	Sleep study	1 (0.7)
Duration of medical procedures	< 15 min	6 (4.2)
	15–30 min	12 (8.3)
	30–60 min	13 (9.0)
	60–120 min	8 (5.6)
	> 120 min or multi sessions	13 (9.0)
	Not reported/ Unclear	97 (67.4)
Types of Procedural Support*	Visit Preparation and Support	61 (42.4)
	Pharmacological Agents	48 (33.3)
	Patient Care Plan or Pathway	43 (29.9)
	Hospital-wide Strategy	19 (13.2)
	Technology (VR)	16 (11.1)
	Behavioural Intervention (i.e. ABA/Visual support/modification techniques/gradual exposure therapy/positive reinforcement))	18 (12.5)
	Environmental Modification (i.e. low stimulus environment)	16 (11.1)
	Distraction (i.e. audio-visual)	13 (9.0)
	Play-based	9 (6.3)
	Communication Support (i.e. ACC, personalised pain communication tool)	4 (2.8)
	Staff Training/Education	4 (2.8)
	Specialist (Nurse navigator, medical clowns, Child Life Specialist)	4 (2.8)
Timing of Procedural Support*	Pre procedure	109 (75.7)
	During procedure	104 (72.2)
	Post procedure	11 (7.6)
	Not applicable	6 (4.2)

*Can have multiple options

### Support Strategies

Our scoping review identified a range of procedural support strategies, with varying implementation across clinical settings and procedural phases. These strategies can be categorised based on their timing (pre-procedure, during procedure, post-procedure) and approach type.

Pre-procedural Support Strategies (n = 110, 76.3%) were most common, with Visit Preparation and Support (n = 61, 42.4%) being the most prevalent strategy, providing structured familiarisation before medical procedures. Pre-procedural Support Strategies were frequently implemented, with Visit Preparation and Support (n = 30) representing a common approach. This included visual pedagogy using photographs, videos, and social stories (n = 11), structured desensitisation and stepwise introduction to clinical environments (n = 8), personalised preparation packages with procedure-specific information (n = 8), mock simulations and rehearsals for procedures (n = 7), and digital applications or multimedia tools for familiarisation (n = 6). Patient Care Plans and Pathways (n = 25) involved individualised planning based on patient-specific needs, including collaborative development with parents/caregivers to identify triggers and preferences (n = 11), documentation of communication methods and sensory needs (n = 8), and scheduling adaptations such as first appointments and reduced waiting times (n = 7). These care plans also incorporated formal risk stratification systems (n = 5), integration of child life specialists for preparation and support (n = 6), standardised templates in electronic medical records (n = 4), and implementation of sensory pathways with dedicated toolkits (n = 3). Additional pre-procedural approaches included Behavioural Interventions for preparation (n = 17) with systematic desensitisation protocols (n = 6), visual aids (n = 7), and Tell-Show-Do techniques (n = 3), as well as Technology-Based Preparation (n = 14) using video modelling (n = 6) and tablet-based visual supports (n = 6).

During-procedure Support Strategies (n = 103, 71.5%) were also frequently implemented, with Pharmacological Agents (n = 48, 33.3%) were used across diverse procedure types with multiple administration routes. Dexmedetomidine emerged as the most versatile agent, administered intranasally (1–4 μg/kg) for radiological/diagnostic procedures (n = 4), dental procedures (n = 2), and needle-related procedures (n = 3), intravenously (0.5–2 μg/kg) primarily for radiological/diagnostic procedures (n = 6), and intramuscularly (4 μg/kg) for emergency and radiological settings (n = 1 each). Midazolam was the most frequently utilised medication across multiple routes: oral administration (0.25–0.5 mg/kg) for dental (n = 4), radiological/diagnostic (n = 5), and needle-related procedures (n = 3); intravenous delivery (0.05–0.45 mg/kg) primarily for radiological/diagnostic procedures (n = 6) and emergency situations (n = 1); and intranasal application (0.2–0.5 mg/kg) across procedures including dental, radiological/diagnostic, needle-related, emergency, and surgical contexts.

General anaesthesia was commonly employed through inhalational approaches for dental (n = 4) and surgery (n = 2), total IV methods for radiological/diagnostic procedures (n = 5), dental (n = 2), and surgery (n = 2), with unspecified protocols primarily used for dental procedures (n = 8). Propofol was administered intravenously as bolus dosing (0.5–3 mg/kg) for radiological/diagnostic procedures (n = 6) and surgery (n = 1), and as continuous infusion for radiological/diagnostic procedures (n = 3). Ketamine demonstrated broad applicability across IV, IM, oral, intranasal, and rectal routes for radiological/diagnostic, dental, and needle-related procedures, while nitrous oxide (50–70%) was frequently used for dental (n = 5), needle-related (n = 4), and radiological/diagnostic procedures (n = 3).

Environmental Modifications (n = 16, 11.1%) addressed sensory sensitivities through reduced stimulation (dimmed lights, minimised noise, limited personnel) (n = 9), sensory-friendly tools (weighted blankets, noise-cancelling headphones) (n = 6), and specialised equipment (mock scanners, adapted dental chairs) (n = 3). Additional environmental adaptations included creating dedicated sensory-adapted environments for specific procedures (n = 4), using projections and visual distractions on walls and ceilings (n = 3), and implementing camouflage techniques for medical equipment (n = 2). Other during-procedure strategies included Behavioural Interventions (n = 18, 12.4%) with Applied Behaviour Analysis techniques (n = 4), graduated exposure therapy prior to the procedure (n = 8), and positive reinforcement strategies (n = 10); Technology-Based Approaches (n = 14) incorporating virtual reality distraction (n = 5), audiovisual distraction using video eyewear (n = 3), and robotic systems for engagement (n = 2); Distraction Techniques (n = 13, 9.0%) including audiovisual methods (n = 8) and music-based strategies (n = 4); and Communication Support (n = 4, 2.8%) using AAC tools (n = 3), personalised pain communication tools (n = 1), and therapeutic communication techniques (n = 1).

Post-Procedure Support Strategies (n = 11, 7.6%) were limited, representing a significant gap in the literature, with only dedicated postoperative care adaptations (n = 2), positive reinforcement for procedure completion (n = 1), and minimised sensory stimuli during recovery (n = 2) identified.

The distribution of support strategies varied across clinical specialties (Supplementary Table 2). Dental settings predominantly implemented visit preparation (n = 19, 13.2%), pharmacological agents (n = 15, 9.7%), and behavioural interventions (n = 13, 9.0%). Medical/Surgical specialties favoured patient care plans (n = 19, 13.2%), visit preparation (n = 15, 10.4%), and pharmacological agents (n = 13, 9.0%). Perioperative settings utilised patient care plans (n = 11, 7.6%) and visit preparation (n = 9, 6.2%), while Emergency departments primarily employed hospital-wide strategies (n = 5, 3.5%) and pharmacological agents (n = 5, 3.5%). Radiology departments most commonly used visit preparation (n = 7, 4.8%) and patient care plans (n = 4, 2.8%).

**Fig. 3:**
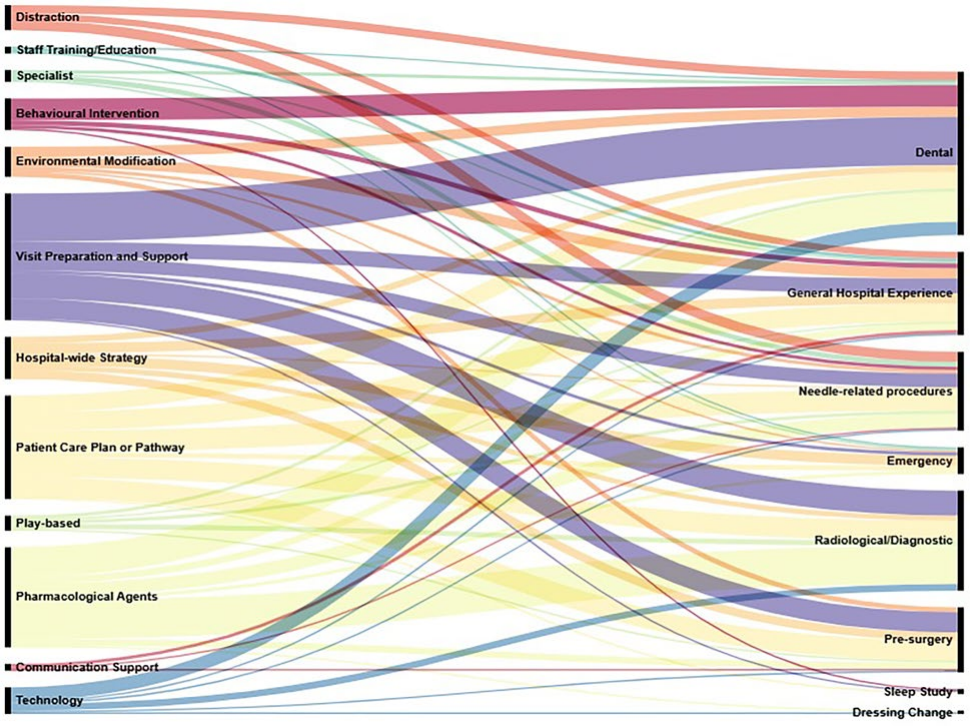
Procedure supports studied across procedure types*, *This alluvial diagram illustrates the distribution of procedural support strategies (left) across the types of medical procedure (right) for children with neurodivergent conditions. The width of each coloured flow represents frequency

Figure 3 depicts procedural support strategies utilised across types of medical procedures. Dental (n = 61; 42.4%) and Radiology/Diagnostic (n = 40; 27.8%) procedures employed the widest variety of supports. Visit Preparation and Support was most frequently implemented (n = 61, 42.4%), particularly in Dental and Radiology/Diagnostic settings. Patient Care Plans/Pathways (n = 43, 29.9%) and Pharmacological Agents (n = 48, 33.3%) were also widely utilised, with the latter predominantly in Radiology/Diagnostic and Dental procedures. Behavioural Interventions (n = 18, 12.5%) were most common in Dental settings, while Hospital-wide Strategies (n = 19, 13.2%) were primarily implemented in Emergency departments. Technology-based approaches, Environmental Modifications, and Distraction techniques were moderately represented, while Communication Support, Staff Training, and Specialist Support were each reported in only 4 studies (2.8%), indicating potential implementation gaps requiring further development.

**Fig. 4:**
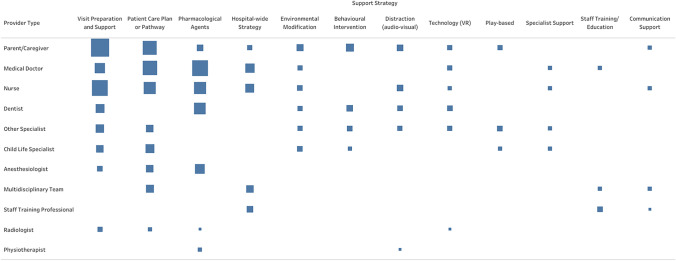
Provider of procedure supports included in the review

Procedural support strategies showed diverse provider involvement (Fig. 4 and Supplementary Table 3). Parents/caregivers primarily implemented visit preparation (n = 41) and participated in care planning (n = 24). Medical doctors mainly administered pharmacological agents (n = 31) and developed care plans (n = 26). Nurses focused on visit preparation (n = 30) and pharmacological support (n = 18).

### Pain and Distress Assessment

As reported in Table 4, pain assessment was limited across studies. Only 13 studies (9%) reported any pain measurement, with observer-assessed pain in 7 studies (4.9%), child-reported pain in 6 studies (4.2%), and parent-reported pain in 6 studies (4.2%). The vast majority (n = 131, 91%) did not report any pain assessment. When pain was assessed, tools included the FLACC scale (n = 3, 2.1%), Wong-Baker FACES Pain Scale (n = 2, 1.4%), Visual Analogue Scale (n = 3, 2.1%), Numeric Rating Scale (n = 3, 2.1%), Faces Pain Scale-Revised (n = 2, 1.4%), Non-Communicating Children’s Pain Checklist (n = 2, 1.4%), Individualised Numeric Rating Scale (n = 1, 0.7%) and Coloured Analogue Scale (n = 1, 0.7%). This highlights a significant gap in pain assessment for neurodivergent children undergoing medical procedures.

**Table 4:** Outcomes reported by authors (N = 144)

Characteristics		N (%)
Pain Assessed by*	Not Applicable/Not Used	131 (91)
	Observer (Clinician/Researcher)	7 (4.9)
	Child	6 (4.2)
	Parent	6 (4.2)
Pain Assessment Tool*	Not Used	131 (91)
	FLACC	3 (2.1)
	Visual Analogue Scale	3 (2.1)
	Numeric Rating Scale	3 (2.1)
	Wong-Baker FACES Pain Scale	2 (1.4)
	Faces Pain Scale-Revised	2 (1.4)
	Non-Communicating Children’s Pain Checklist	2 (1.4)
	Individualised Numeric Rating	1 (0.7)
	Qualitative: Talking Mat and Parent Interviews	1 (0.7)
	Coloured Analogue Scale	1 (0.7)
Distress assessed by*	Observer (Clinician/Researcher)	97 (67.4)
	Not Applicable/Not Used	37 (25.7)
	Parent	40 (27.8)
	Child	6 (4.2%)
Distress Assessment Tool*	Not used	37 (25.7)
	Qualitative (interviews, observation, surveys)	37 (25.7)
	Behavioural/Clinical Observation (e.g. cooperation)	32 (22.2)
	Frankl Behaviour Scale	13 (9.0)
	Physiological Measures	11 (7.6)
	Motion Compliance (Successful completion of Scan/test required immobilisation)	9 (6.3)
	Numerical Rating Scale/ Visual Analogue Scale	7 (4.86)
	Venham’s Anxiety/ Behavioural Rating Scale	5 (3.5)
	State-Trait Anxiety Inventory	3 (2.1)
	Observational Scale of Behavioural Distress	3 (2.1)
	Children’s Dental Behaviour Rating Scale	2 (1.4)
	Video Analysis	2 (1.4)
	Child Fear Scale	1 (0.7)
	Visual Facial Anxiety Scale	1 (0.7)
	Children’s Emotional Manifestation Scale	1 (0.7)
	Child Induction Behavioral Assessment Scale	1 (0.7)
	Child–Adult Medical Procedure Interaction Scale	1 (0.7)
	Psychosocial Risk Assessment in Pediatrics	1 (0.7)

*FLACC* Face, Legs, Activity, Cry, Consolability scale

*Can have multiple options

Distress assessment was more commonly reported, with 67.4% (n = 97) of studies including observer-assessed distress, 27.8% (n = 40) including parent-assessed distress, and only 4.2% (n = 6) including child-assessed distress. Forty-three per cent (n = 62) of papers only assessed observer-reported distress. Assessment methods varied widely, with a substantial proportion (n = 69, 47.9%) not using any formal distress assessment tool. Among those that did use formal assessment methods, qualitative approaches (interviews, observation, surveys) were most common (n = 37, 25.7%), followed by behavioural/clinical observation (n = 32, 22.2%), the Frankl Behaviour Scale (n = 13, 9.0%), and physiological measures (n = 11, 7.6%).

### Outcomes of Procedural Support

Of the 144 included studies, most (n = 125, 86.8%) reported a global judgement of satisfaction with procedural support, although this was based on subjective impressions. Satisfaction was rated as positive in 94 studies (65.3%), mixed in 30 studies (20.8%), and no effect in 1 study (0.7%). The remaining studies (n = 19, 13.2%) provided non-evaluative or not applicable responses. Adverse events were reported in 40 studies (27.8%), primarily with pharmacological interventions (n = 26). Common complications included cardiovascular and respiratory effects and gastrointestinal symptoms. Non-pharmacological strategies had fewer adverse events, with visit preparation occasionally resulting in anxiety or procedural rescheduling (n = 6), and physical restraint associated with patient distress (n = 3).

### Barriers of Procedural Support Implementation

Across the dataset, 129 out of 144 studies (89.6%) reported specific barriers encountered in the implementation of support strategies for neurodivergent individuals undergoing healthcare procedures. These barriers ranged from systemic issues, like limited ASD-specific training among healthcare providers, resource shortages, and lack of standardised protocols, to patient-centred factors such as sensory sensitivities, communication difficulties, or behavioural challenges specific to neurodivergent populations. A recurrent theme in these barriers is the gap in training and preparedness among healthcare professionals. Many studies pointed out that clinicians often feel underprepared to adequately assess and manage the unique needs of neurodivergent patients, leading to suboptimal care experiences. Environmental factors, such as overstimulating clinical settings, also frequently emerged as obstacles, indicating the need for sensory-friendly modifications. It signals that while individual interventions are being designed, the broader infrastructure and clinician competencies still lag behind the ideal standards for inclusive care.

### Facilitators of Procedural Support Implementation

A total of 143 studies (99.3%) reported facilitators or potential facilitators of procedural support implementation. Healthcare provider training was consistently reported as a key facilitator, with authors describing how formal education programs in ASD and broader neurodivergent conditions directly addressed implementation barriers. Studies highlighted programs that enhanced healthcare workers’ competencies in specialised communication approaches, behavioural management techniques, and recognition of sensory sensitivities.

Family-centred care emerged as another significant facilitator identified by the authors. Multiple studies reported that engaging parents or caregivers through systematic processes, including advanced disclosure of patient needs, collaborative care planning sessions, and parent presence during procedures, helped ease anxiety for patients and improved procedural compliance. Authors noted that parents often provided critical insights about individual triggers, effective calming strategies, and communication preferences that would otherwise be inaccessible to healthcare teams. Authors of several studies emphasised that facilities implementing multi-component approaches, integrating staff training, family collaboration, and environmental adaptations, reported more consistent positive outcomes than those utilising single-strategy interventions.

## Discussion

The scoping review of 144 studies provides a comprehensive overview of the current evidence on procedural support strategies for neurodivergent children. Our findings highlight several important gaps and trends in the existing literature, revealing both promising approaches and significant shortcomings in research methodology, population representation, and assessment practices. Most studies reported positive outcomes from interventions, though these were primarily based on subjective impressions rather than rigorous measurement.

### Research Focus and Gaps

A key finding is the striking research imbalance across neurodivergent diagnoses. The predominance of studies focusing on children with ASD (n = 121, 84.0%) compared to other neurodivergent conditions represents a significant gap in the literature. Children with ADHD (n =302, 20.8%), communication disorders (n = 5, 3.5%), and specific learning disorders (n = 1, 0.7%) are particularly underrepresented, despite these conditions affecting substantial numbers of children globally (Harris et al., 2024; Johnson et al., 2023). This disproportionate focus limits our understanding of how different neurodivergent conditions might necessitate tailored procedural support approaches. The high proportion of studies conducted in dental settings (n = 50, 34.7%) provides valuable insights for dental procedures but may not translate directly to other medical contexts. The limited research in emergency departments (n = 10 , 6.9%) is particularly concerning, as these often involve urgent, unfamiliar procedures that can be especially challenging for neurodivergent children (Dwyer & Rogan, 2022; Rotella, 2022).

### Procedural Support Strategies

Our scoping review identified a range of procedural support strategies, with varying implementation across clinical settings and procedural phases. Visit preparation and support emerged as the most common strategy, aligning with previous research highlighting the importance of predictability and preparation for neurodivergent children (Johnson et al., 2023; Leblanc et al., 2024). However, the effectiveness of these approaches was typically evaluated through global satisfaction judgments or unstandardized assessments rather than validated outcome measures. These strategies included visual pedagogy using photographs, videos, and social stories (n = 11), structured desensitisation and stepwise introduction to clinical environments (n = 17), personalised preparation packages with procedure-specific information (n = 12), mock simulations and rehearsals for procedures (n = 10), and digital applications or multimedia tools for familiarisation (n = 7). The minimal use of communication support strategies (n = 3, 2.1%) is striking, given the communication challenges many neurodivergent children face (Bond et al., 2025). This included augmentative and alternative communication (AAC) tools (n = 3), personalised pain communication tools (n = 1), therapeutic communication techniques (n = 1), and direct communication adaptations for individual needs (n = 2). This suggests a critical need for research on augmentative and alternative communication approaches during medical procedures (Bond et al., 2025). Similarly, while environmental modifications (n = 16, 11.1%) were implemented in some studies, including reduced stimulation environments (n = 9), sensory-friendly tools (n = 6), and specialised equipment adaptations (n = 3), this represents a relatively underutilised approach. Given that sensory sensitivities are common among neurodivergent children, more extensive implementation of sensory-adapted environments may help address these needs through thoughtful environmental design (Talley et al., 2024).

### Pain and Distress Assessment: Challenges and Barriers

While not all procedures included in this review were inherently painful (e.g., non-invasive diagnostic imaging, dental examinations without treatment), the widespread inadequacy of pain assessment even in clearly painful procedures represents a significant gap. The fact that 91% of studies did not report any pain assessment highlights a fundamental gap in procedural care for neurodivergent children. When assessment was reported, it rarely incorporated the child’s perspective (only n = 6, 4.2% of studies), despite mounting evidence of the importance of self-report in pain assessment whenever possible (Herr et al., 2024; Neshat & Ghorbani, 2023; Zontag et al., 2022). This is particularly concerning given that neurodivergent children may experience and express pain differently due to sensory processing differences, communication challenges, and atypical pain behaviours (Neshat & Ghorbani, 2023; Zontag et al., 2022). The reliance on observer-reported distress (n = 97, 67.4%), typically through unstructured clinical observation rather than validated tools, raises concerns about the reliability and validity of these assessments (Herr et al., 2024). Prospective cohort studies comparing pain scales in youth with physical disabilities has shown that not all scales perform equally well; for example, the 0–10 Numerical Rating Scale (NRS-11) has demonstrated superior validity compared to the Wong-Baker FACES Pain Rating Scale and Verbal Rating Scales in youth with physical disabilities (Johnson et al., 2023; Miró et al., 2016) Only six studies utilised tools tailored specifically for moderate to severe cognitive impairment or non-verbal children; FLACC (n = 2), Non-communicating children’s pain checklist (n = 2), Revised-FLACC (n = 1), and INRS (n = 1) – none of which assess distress or are child-reported (Breau et al., 2002; Crosta et al., 2014; Malviya et al., 2006). Revised-FLACC and INRS are promising, as they are individualised to the child’s unique pain behaviours as identified by the caregiver. This represents a significant research gap, likely as the development of validated and accurate self-reported pain and distress assessment tools poses a considerable challenge in this population. Additionally, the validity of certain scales may be age-dependent, with some measures functioning differently across developmental stages (Johnson et al., 2023). This highlights the need for selecting developmentally appropriate and validated pain assessment tools for neurodivergent children, with special consideration for those with communication challenges who may benefit from adapted self-report measures like the Non-Communicating Children’s Pain Checklist or visually simplified analog scales (Breau et al., 2002; Crosta et al., 2014; Malviya et al., 2006; Miró et al., 2016).

### Methodological Considerations

The methodological quality of included studies varied considerably. While the inclusion of qualitative research (n = 26, 18.1%) provides valuable insights into the experiences of neurodivergent children and their families, the relative scarcity of high-quality randomised controlled trials (n = 20, 13.9%) limits our ability to draw firm conclusions about intervention effectiveness. However, RCTs may not always be the most appropriate or feasible design for studying neurodivergent populations, where high individual variability, ethical considerations around withholding individualised supports, and the need for personalisation often favour alternative methodologies, including qualitative research, pragmatic trials, and single-case designs. As the complexities of accurately assessing the atypical processing and expression of pain and distress, particularly in the more severely cognitively impaired paediatric population, it is recommended that an individualised and mixed-method approach is taken, with a combination of self-reported, caregiver-reported and observer-reported (i.e. physiological measures) validated tools (Albani et al., 2022). The predominance of global satisfaction judgments rather than structured outcome measures further complicates the evaluation of different support strategies. Future research would benefit from more rigorous study designs with clearly defined intervention protocols, adequate sample sizes, appropriate control or comparison groups, and standardised outcome measures validated for neurodivergent populations.

## Limitations

Several limitations should be considered when interpreting these findings. First, the restriction to English-language publications published between 2014–2025 may have excluded relevant studies. Second, the heterogeneity of neurodivergent conditions, procedural contexts, and outcome measures makes direct comparisons challenging. Finally, while the scoping methodology was appropriate for comprehensively mapping this diverse field, it has inherent limitations. Scoping reviews prioritise breadth of evidence mapping over depth of synthesis and therefore do not include formal quality assessment of individual studies or quantitative synthesis of intervention effectiveness. Future systematic reviews, informed by the research gaps identified in this scoping work, will be necessary to evaluate intervention effectiveness for specific populations or procedure types rigorously and to develop evidence-based clinical practice recommendations.

Despite these limitations, several potential practical implications emerge from this review. The diversity of neurodivergent conditions necessitates individualised assessment and support strategies, with pre-procedure planning appearing particularly important for adapting to each child’s specific needs and preferences. Based on reported outcomes, approaches that combine multiple support strategies (such as preparation, environmental modification, and appropriate pharmacological support when necessary) appeared to be more favourably received than single interventions, though this was typically based on subjective impressions rather than rigorous comparative evaluation. Parent/caregiver involvement in pre-procedural planning was a common theme across successful interventions with reported positive outcomes, highlighting the potential importance of leveraging families’ expertise about their child’s specific needs and responses. The limited focus on staff training (n = 4, 2.8% of studies) represents a gap that could be addressed in practice through increased education about neurodivergent children’s needs during medical procedures.

## Future Research Directions

This review identifies key priorities for future research to advance procedural support for neurodivergent children. First, there is an urgent need for studies that include neurodivergent populations beyond autism, particularly children with ADHD, communication disorders, intellectual disability and learning disorders, who are currently underrepresented in the literature. Future research should also examine how intersecting identities, including gender, race, ethnicity, socioeconomic status, and cultural background, influence procedural experiences for neurodivergent children, as the current literature rarely addresses the compounded barriers faced by those from minoritised communities. Additionally, the development and validation of pain and distress assessment tools tailored to the needs of neurodivergent children, especially those with communication challenges, should be prioritised to enable accurate and meaningful outcome measurement. Child-centred research approaches are also critical; incorporating the perspectives of neurodivergent children themselves through inclusive research approaches and appropriately adapted self-report measures would provide valuable insights that are largely absent from existing evidence. Comparative effectiveness trials are needed to evaluate different procedural support strategies, including non-pharmacological interventions, to determine what works best for specific procedures and populations. Finally, implementation science should play a greater role in future research, with a focus on understanding and overcoming the practical barriers to applying evidence-based strategies across diverse healthcare settings, thereby supporting their integration into routine clinical practice.

## Conclusion

Despite approximately 1 in 6 children in high-income countries being diagnosed with neurodivergent conditions, this scoping review highlighted gaps in the evidence base for procedural support strategies for this vulnerable population. While potentially promising approaches exist, particularly related to preparation and individualised support, rigorous evaluation of their effectiveness is limited by inadequate assessment methods, narrow population focus, and methodological challenges. Most reported outcomes relied on subjective impressions rather than validated measures, limiting conclusions about intervention effectiveness. Future research addressing these gaps has the potential to substantially improve the procedural experiences and outcomes for this vulnerable population.

## Supplementary Information

Below is the link to the electronic supplementary material.

Supplementary Material 1

## Data Availability

The datasets supporting the conclusions of this article are included within the article and its supplementary materials. The full dataset and data extraction forms are available from the corresponding author upon reasonable request.
