# Supplementary material for: Procedural Support for Neurodivergent Children During Medical Procedures: A Scoping Review
Source: Clin Child Fam Psychol Rev. 2026 Feb 24;29(1):167–81. doi: 10.1007/s10567-026-00562-w (PMC12979319; doi:10.1007/s10567-026-00562-w)
Supplement: Supplementary file 1 — Supplementary Material 1 [file 10567_2026_562_MOESM1_ESM.docx]

**Supplementary Material 1: Database Searches**

**Date searched:** 10 January 2025

**PubMed search** 3761 results

Includes MeSH

**(**"Neurodevelopmental Disorders"[Mesh] OR "Communication Disorders"[Mesh] OR "Intellectual Disability"[Mesh] OR “neurodevelopmental”[tiab] OR “neurodivergent”[tiab] OR “neurodiversity”[tiab] OR “neurodiverse”[tiab] OR “autism”[tiab] OR “autistic”[tiab] OR “Asperger’s”[tiab] OR “Aspergers”[tiab] OR “Asperger”[tiab] OR “Aspergic”[tiab] OR “ASD”[tiab] OR “attention deficit hyperactivity disorder”[tiab] OR “attention deficit hyperactivity disorders”[tiab] OR “attention-deficit/hyperactivity disorder”[tiab] OR “attention-deficit/hyperactivity disorders”[tiab] OR “attention deficit-hyperactivity disorder”[tiab] OR “attention deficit-hyperactivity disorders”[tiab] OR “ADHD”[tiab] OR “ADDH”[tiab] OR “attention deficit disorder”[tiab] OR “attention deficit disorders”[tiab] OR “ADD”[tiab] OR “developmental disorder”[tiab] OR “developmental disorders”[tiab] OR “developmental disability”[tiab] OR “developmental disabilities”[tiab] OR “childhood disintegrative disorder”[tiab] OR “intellectual disabilities”[tiab] OR “intellectual disability”[tiab] OR “development disorder”[tiab] OR “development disorders”[tiab] OR “intellectual disorders”[tiab] OR “intellectual disorder”[tiab] OR “communication disorders”[tiab] OR “communication disorder”[tiab] OR “communicative disorder”[tiab] OR “communicative disorders”[tiab] OR “communication disabilities”[tiab] OR “communication disability”[tiab] OR “communicative dysfunction”[tiab] OR “communicative dysfunctions”[tiab] OR “language disorders”[tiab] OR “language disorder”[tiab] OR “language impairment”[tiab] OR “language impairments”[tiab] OR “learning disorder”[tiab] OR “learning disorders”[tiab] OR “learning disability”[tiab] OR “learning disabilities”[tiab] OR “learning disturbance”[tiab] OR “learning disturbances”[tiab] OR “speech sound disorder”[tiab] OR “speech sound disorders”[tiab] OR “childhood-onset fluency disorder”[tiab] OR “childhood-onset fluency disorders”[tiab] OR “childhood onset fluency disorders”[tiab] OR “childhood onset fluency disorders”[tiab] OR “stuttering”[tiab] OR “phonological disorder”[tiab] OR “phonological disorders”[tiab] OR “mental retardation”[tiab] OR “developmental coordination disorder”[tiab] OR “developmental coordination disorders”[tiab] OR “stereotypic movement disorder”[tiab] OR “stereotypic movement disorders”[tiab] OR “tic disorder”[tiab] OR “tic disorders”[tiab] OR “Tourette’s”[tiab] OR “Tourette”[tiab] OR “Tourettes”[tiab] OR “dyslexia”[tiab] OR “dyslexias”[tiab] OR “dyslexic”[tiab] OR “dyslexics”[tiab] OR “dysgraphia”[tiab] OR “dysgraphias”[tiab] OR “agraphia”[tiab] OR “agraphias”[tiab] OR “dyscalculia”[tiab] OR “dyscalculias”[tiab] OR “acalculia”[tiab] OR “acalculias”[tiab] OR “reading disorder”[tiab] OR “reading disorders”[tiab] OR “reading disability”[tiab] OR “reading disabilities”[tiab] OR “mathematics disorder”[tiab] OR “mathematics disorders”[tiab] OR “disorder of written expression”[tiab] OR “written expression disorder”[tiab] OR “written expression disorders”[tiab] OR “sensory processing disorder”[tiab] OR “sensory processing disorders”[tiab] OR “auditory processing disorder”[tiab] OR “auditory processing disorders”[tiab] OR “visual processing disorder”[tiab] OR “visual processing disorders”[tiab] OR “dyspraxia”[tiab] OR “dyspraxias”[tiab] OR “developmental coordination disorder”[tiab]**) AND (**"Child"[Mesh] OR "Infant"[Mesh] OR "Adolescent"[Mesh] OR "Pediatrics"[Mesh] OR "Pediatric Nursing"[Mesh] OR "Nurses, Pediatric"[Mesh] OR "Pediatric Nurse Practitioners"[Mesh] OR "Hospitals, Pediatric"[Mesh] OR "Pediatric Emergency Medicine"[Mesh] OR "Pediatric Anesthesia"[Mesh] OR "Adolescent Medicine"[Mesh] OR "Adolescent, Hospitalized"[Mesh] OR "Adolescent Health Services"[Mesh] OR "Child Health Services"[Mesh] OR “child”[tiab] OR “children”[tiab] OR “infant”[tiab] OR “infants”[tiab] OR “baby”[tiab] OR “babies”[tiab] OR “toddler”[tiab] OR “toddlers”[tiab] OR “adolescent”[tiab] OR “adolescents”[tiab] OR “adolescence”[tiab] OR “youth”[tiab] OR “youths”[tiab] OR “teen”[tiab] OR “teens”[tiab] OR “teenager”[tiab] OR “teenagers”[tiab] OR “young person”[tiab] OR “young people”[tiab] OR “pediatric”[tiab] OR “pediatrics”[tiab] OR “paediatric”[tiab] OR “paediatrics”[tiab]**) AND (**“procedural”[tiab] OR “procedure”[tiab] OR “procedures”[tiab] OR “imaging”[tiab] OR “radiological”[tiab] OR “radiology”[tiab] OR “surgical”[tiab] OR “needle”[tiab] OR “needles”[tiab] OR “venipuncture”[tiab] OR “venepuncture”[tiab] OR “healthcare encounters”[tiab] OR “healthcare encounter”[tiab] OR “hospital stay”[tiab] OR “hospital stays”[tiab] OR “hospital visit”[tiab] OR “hospital visits”[tiab] OR “hospitalisation”[ti] OR “hospitalisations”[ti] OR “hospitalization”[ti] OR “hospitalizations”[ti] OR “hospitalised”[ti] OR “hospitalized”[ti] OR “presentations”[ti] OR “presentations”[ti] OR “pain”[ti]**) AND (**(“support”[tiab] OR “supportive”[tiab] OR “care”[tiab] OR “adjustments”[tiab] OR “adjustment”[tiab] OR “accommodation”[tiab] OR “accommodations”[tiab] OR “special considerations”[tiab] OR “special needs”[tiab] OR “additional needs”[tiab] OR “individual needs”[tiab]) **OR (**(“intervention”[tiab] OR “interventions”[tiab] OR “management”[tiab] OR “procedural”[tiab] OR “strategies”[tiab] OR “strategy”[tiab] OR “technique”[tiab] OR “techniques”[tiab] OR “approach”[tiab] OR “approaches”[tiab] OR “protocol”[tiab] OR “clinical pathway”[tiab] OR “undergoing”[tiab]) **AND** (“behaviour”[tiab] OR “behavioural”[tiab] OR “psychological”[tiab] OR “hypnosis”[tiab] OR “distraction”[tiab] OR “diversion”[tiab] OR “diversional”[tiab] OR “pharmacological”[tiab] OR “sedation”[tiab] OR “sedative”[tiab] OR “sedatives”[tiab] OR “sedate”[tiab] OR “sedates”[tiab] OR “sedated”[tiab] OR “sedating”[tiab] OR “analgesic”[tiab] OR “analgesics”[tiab] OR “anaesthetic”[tiab] OR “anaesthetics”[tiab] OR “anesthetic”[tiab] OR “anesthetics”[tiab] OR “anaesthesia”[tiab] OR “anesthesia”[tiab] OR “physical”[tiab] OR “heat”[tiab] OR “cold”[tiab] OR “buzzy”[tiab] OR “vibration”[tiab] OR “restraint”[tiab] OR “restraints”[tiab] OR “child-focused”[tiab] OR “child focused”[tiab] OR “child-centred”[tiab] OR “child centred”[tiab] OR “child-centered”[tiab] OR “child centered”[tiab])**)) AND (**eng[la] OR und[la]**) AND** 2014:2025[dp] **NOT** **(**animals [mh] NOT humans [mh]**)**

**Embase (Elsevier)** 6393 results to 3950

#1 AND (**'article'**/it OR **'article in press'**/it OR **'preprint'**/it OR **'review'**/it)

[3,950](https://www.embase.com/)

Includes Emtree

**(**'attention deficit hyperactivity disorder'/exp/mj OR 'autism'/exp/mj OR 'developmental disorder'/exp/mj OR 'disorders of higher cerebral function'/exp/mj OR 'behavior disorder'/exp/mj OR 'learning disorder'/exp/mj OR 'motor dysfunction'/exp/mj OR 'developmental coordination disorder'/exp/mj OR 'tic'/exp/mj OR 'Gilles de la Tourette syndrome'/exp/mj OR 'speech disorder'/exp/mj OR 'communication disorder'/exp/mj OR 'intellectual impairment'/exp/mj OR 'neurodiverse person'/exp/mj OR 'neurodiversity'/exp/mj OR “neurodevelopmental”:ti,ab OR “neurodivergent”:ti,ab OR “neurodiversity”:ti,ab OR “neurodiverse”:ti,ab OR “autism”:ti,ab OR “autistic”:ti,ab OR “Asperger s”:ti,ab OR “Aspergers”:ti,ab OR “Asperger”:ti,ab OR “Aspergic”:ti,ab OR “ASD”:ti,ab OR “attention deficit hyperactivity disorder”:ti,ab OR “attention deficit hyperactivity disorders”:ti,ab OR “attention-deficit/hyperactivity disorder”:ti,ab OR “attention-deficit/hyperactivity disorders”:ti,ab OR “attention deficit-hyperactivity disorder”:ti,ab OR “attention deficit-hyperactivity disorders”:ti,ab OR “ADHD”:ti,ab OR “ADDH”:ti,ab OR “attention deficit disorder”:ti,ab OR “attention deficit disorders”:ti,ab OR “ADD”:ti,ab OR “developmental disorder”:ti,ab OR “developmental disorders”:ti,ab OR “developmental disability”:ti,ab OR “developmental disabilities”:ti,ab OR “childhood disintegrative disorder”:ti,ab OR “intellectual disabilities”:ti,ab OR “intellectual disability”:ti,ab OR “development disorder”:ti,ab OR “development disorders”:ti,ab OR “intellectual disorders”:ti,ab OR “intellectual disorder”:ti,ab OR “communication disorders”:ti,ab OR “communication disorder”:ti,ab OR “communicative disorder”:ti,ab OR “communicative disorders”:ti,ab OR “communication disabilities”:ti,ab OR “communication disability”:ti,ab OR “communicative dysfunction”:ti,ab OR “communicative dysfunctions”:ti,ab OR “language disorders”:ti,ab OR “language disorder”:ti,ab OR “language impairment”:ti,ab OR “language impairments”:ti,ab OR “learning disorder”:ti,ab OR “learning disorders”:ti,ab OR “learning disability”:ti,ab OR “learning disabilities”:ti,ab OR “learning disturbance”:ti,ab OR “learning disturbances”:ti,ab OR “speech sound disorder”:ti,ab OR “speech sound disorders”:ti,ab OR “childhood-onset fluency disorder”:ti,ab OR “childhood-onset fluency disorders”:ti,ab OR “childhood onset fluency disorders”:ti,ab OR “childhood onset fluency disorders”:ti,ab OR “stuttering”:ti,ab OR “phonological disorder”:ti,ab OR “phonological disorders”:ti,ab OR “mental retardation”:ti,ab OR “developmental coordination disorder”:ti,ab OR “developmental coordination disorders”:ti,ab OR “stereotypic movement disorder”:ti,ab OR “stereotypic movement disorders”:ti,ab OR “tic disorder”:ti,ab OR “tic disorders”:ti,ab OR “Tourette s”:ti,ab OR “Tourette”:ti,ab OR “Tourettes”:ti,ab OR “dyslexia”:ti,ab OR “dyslexias”:ti,ab OR “dyslexic”:ti,ab OR “dyslexics”:ti,ab OR “dysgraphia”:ti,ab OR “dysgraphias”:ti,ab OR “agraphia”:ti,ab OR “agraphias”:ti,ab OR “dyscalculia”:ti,ab OR “dyscalculias”:ti,ab OR “acalculia”:ti,ab OR “acalculias”:ti,ab OR “reading disorder”:ti,ab OR “reading disorders”:ti,ab OR “reading disability”:ti,ab OR “reading disabilities”:ti,ab OR “mathematics disorder”:ti,ab OR “mathematics disorders”:ti,ab OR “disorder of written expression”:ti,ab OR “written expression disorder”:ti,ab OR “written expression disorders”:ti,ab OR “sensory processing disorder”:ti,ab OR “sensory processing disorders”:ti,ab OR “auditory processing disorder”:ti,ab OR “auditory processing disorders”:ti,ab OR “visual processing disorder”:ti,ab OR “visual processing disorders”:ti,ab OR “dyspraxia”:ti,ab OR “dyspraxias”:ti,ab OR “developmental coordination disorder”:ti,ab**) AND (**'child'/exp/mj OR 'adolescent'/exp/mj OR 'pediatrics'/exp/mj OR 'pediatric nursing'/exp/mj OR 'pediatric nurse'/exp/mj OR 'pediatric nurse practitioner'/exp/mj OR 'pediatric hospital'/exp/mj OR 'pediatric emergency medicine'/exp/mj OR 'pediatric anesthesia'/exp/mj OR 'hospitalized adolescent'/exp/mj OR 'hospitalized child'/exp/mj OR 'hospitalized infant'/exp/mj OR 'child health care'/exp/mj OR “child”:ti,ab OR “children”:ti,ab OR “infant”:ti,ab OR “infants”:ti,ab OR “baby”:ti,ab OR “babies”:ti,ab OR “toddler”:ti,ab OR “toddlers”:ti,ab OR “adolescent”:ti,ab OR “adolescents”:ti,ab OR “adolescence”:ti,ab OR “youth”:ti,ab OR “youths”:ti,ab OR “teen”:ti,ab OR “teens”:ti,ab OR “teenager”:ti,ab OR “teenagers”:ti,ab OR “young person”:ti,ab OR “young people”:ti,ab OR “pediatric”:ti,ab OR “pediatrics”:ti,ab OR “paediatric”:ti,ab OR “paediatrics”:ti,ab**) AND (**“procedural”:ti,ab OR “procedure”:ti,ab OR “procedures”:ti,ab OR “imaging”:ti,ab OR “radiological”:ti,ab OR “radiology”:ti,ab OR “surgical”:ti,ab OR “needle”:ti,ab OR “needles”:ti,ab OR “venipuncture”:ti,ab OR “venepuncture”:ti,ab OR “healthcare encounters”:ti,ab OR “healthcare encounter”:ti,ab OR “hospital stay”:ti,ab OR “hospital stays”:ti,ab OR “hospital visit”:ti,ab OR “hospital visits”:ti,ab OR “hospitalisation”:ti OR “hospitalisations”:ti OR “hospitalization”:ti OR “hospitalizations”:ti OR “hospitalised”:ti OR “hospitalized”:ti OR “presentations”:ti OR “presentations”:ti OR “pain”:ti**) AND (**(“support”:ti,ab OR “supportive”:ti,ab OR “care”:ti,ab OR “adjustments”:ti,ab OR “adjustment”:ti,ab OR “accommodation”:ti,ab OR “accommodations”:ti,ab OR “special considerations”:ti,ab OR “special needs”:ti,ab OR “additional needs”:ti,ab OR “individual needs”:ti,ab) **OR (**(“intervention”:ti,ab OR “interventions”:ti,ab OR “management”:ti,ab OR “procedural”:ti,ab OR “strategies”:ti,ab OR “strategy”:ti,ab OR “technique”:ti,ab OR “techniques”:ti,ab OR “approach”:ti,ab OR “approaches”:ti,ab OR “protocol”:ti,ab OR “clinical pathway”:ti,ab OR “undergoing”:ti,ab) **AND** (“behaviour”:ti,ab OR “behavioural”:ti,ab OR “psychological”:ti,ab OR “hypnosis”:ti,ab OR “distraction”:ti,ab OR “diversion”:ti,ab OR “diversional”:ti,ab OR “pharmacological”:ti,ab OR “sedation”:ti,ab OR “sedative”:ti,ab OR “sedatives”:ti,ab OR “sedate”:ti,ab OR “sedates”:ti,ab OR “sedated”:ti,ab OR “sedating”:ti,ab OR “analgesic”:ti,ab OR “analgesics”:ti,ab OR “anaesthetic”:ti,ab OR “anaesthetics”:ti,ab OR “anesthetic”:ti,ab OR “anesthetics”:ti,ab OR “anaesthesia”:ti,ab OR “anesthesia”:ti,ab OR “physical”:ti,ab OR “heat”:ti,ab OR “cold”:ti,ab OR “buzzy”:ti,ab OR “vibration”:ti,ab OR “restraint”:ti,ab OR “restraints”:ti,ab OR “child-focused”:ti,ab OR “child focused”:ti,ab OR “child-centred”:ti,ab OR “child centred”:ti,ab OR “child-centered”:ti,ab OR “child centered”:ti,ab)**)) AND** [english]/lim **AND** [2014-2025]/py **NOT (**‘animal experiment’/de NOT (‘human experiment’/de OR ‘human’/de)**)** AND (**'article'**/it OR **'article in press'**/it OR **'preprint'**/it OR **'review'**/it)

**CINAHL Complete (EBSCOhost)** 1662 results

Includes CINAHL Subject Headings

**(**MH "Mental Disorders Diagnosed in Childhood+" OR MH "Communicative Disorders+" OR MH "Intellectual Disability+" OR TI(“neurodevelopmental” OR “neurodivergent” OR “neurodiversity” OR “neurodiverse” OR “autism” OR “autistic” OR “Asperger’s” OR “Aspergers” OR “Asperger” OR “Aspergic” OR “ASD” OR “attention deficit hyperactivity disorder” OR “attention deficit hyperactivity disorders” OR “attention-deficit/hyperactivity disorder” OR “attention-deficit/hyperactivity disorders” OR “attention deficit-hyperactivity disorder” OR “attention deficit-hyperactivity disorders” OR “ADHD” OR “ADDH” OR “attention deficit disorder” OR “attention deficit disorders” OR “ADD” OR “developmental disorder” OR “developmental disorders” OR “developmental disability” OR “developmental disabilities” OR “childhood disintegrative disorder” OR “intellectual disabilities” OR “intellectual disability” OR “development disorder” OR “development disorders” OR “intellectual disorders” OR “intellectual disorder” OR “communication disorders” OR “communication disorder” OR “communicative disorder” OR “communicative disorders” OR “communication disabilities” OR “communication disability” OR “communicative dysfunction” OR “communicative dysfunctions” OR “language disorders” OR “language disorder” OR “language impairment” OR “language impairments” OR “learning disorder” OR “learning disorders” OR “learning disability” OR “learning disabilities” OR “learning disturbance” OR “learning disturbances” OR “speech sound disorder” OR “speech sound disorders” OR “childhood-onset fluency disorder” OR “childhood-onset fluency disorders” OR “childhood onset fluency disorders” OR “childhood onset fluency disorders” OR “stuttering” OR “phonological disorder” OR “phonological disorders” OR “mental retardation” OR “developmental coordination disorder” OR “developmental coordination disorders” OR “stereotypic movement disorder” OR “stereotypic movement disorders” OR “tic disorder” OR “tic disorders” OR “Tourette’s” OR “Tourette” OR “Tourettes” OR “dyslexia” OR “dyslexias” OR “dyslexic” OR “dyslexics” OR “dysgraphia” OR “dysgraphias” OR “agraphia” OR “agraphias” OR “dyscalculia” OR “dyscalculias” OR “acalculia” OR “acalculias” OR “reading disorder” OR “reading disorders” OR “reading disability” OR “reading disabilities” OR “mathematics disorder” OR “mathematics disorders” OR “disorder of written expression” OR “written expression disorder” OR “written expression disorders” OR “sensory processing disorder” OR “sensory processing disorders” OR “auditory processing disorder” OR “auditory processing disorders” OR “visual processing disorder” OR “visual processing disorders” OR “dyspraxia” OR “dyspraxias” OR “developmental coordination disorder”) OR AB(“neurodevelopmental” OR “neurodivergent” OR “neurodiversity” OR “neurodiverse” OR “autism” OR “autistic” OR “Asperger’s” OR “Aspergers” OR “Asperger” OR “Aspergic” OR “ASD” OR “attention deficit hyperactivity disorder” OR “attention deficit hyperactivity disorders” OR “attention-deficit/hyperactivity disorder” OR “attention-deficit/hyperactivity disorders” OR “attention deficit-hyperactivity disorder” OR “attention deficit-hyperactivity disorders” OR “ADHD” OR “ADDH” OR “attention deficit disorder” OR “attention deficit disorders” OR “ADD” OR “developmental disorder” OR “developmental disorders” OR “developmental disability” OR “developmental disabilities” OR “childhood disintegrative disorder” OR “intellectual disabilities” OR “intellectual disability” OR “development disorder” OR “development disorders” OR “intellectual disorders” OR “intellectual disorder” OR “communication disorders” OR “communication disorder” OR “communicative disorder” OR “communicative disorders” OR “communication disabilities” OR “communication disability” OR “communicative dysfunction” OR “communicative dysfunctions” OR “language disorders” OR “language disorder” OR “language impairment” OR “language impairments” OR “learning disorder” OR “learning disorders” OR “learning disability” OR “learning disabilities” OR “learning disturbance” OR “learning disturbances” OR “speech sound disorder” OR “speech sound disorders” OR “childhood-onset fluency disorder” OR “childhood-onset fluency disorders” OR “childhood onset fluency disorders” OR “childhood onset fluency disorders” OR “stuttering” OR “phonological disorder” OR “phonological disorders” OR “mental retardation” OR “developmental coordination disorder” OR “developmental coordination disorders” OR “stereotypic movement disorder” OR “stereotypic movement disorders” OR “tic disorder” OR “tic disorders” OR “Tourette’s” OR “Tourette” OR “Tourettes” OR “dyslexia” OR “dyslexias” OR “dyslexic” OR “dyslexics” OR “dysgraphia” OR “dysgraphias” OR “agraphia” OR “agraphias” OR “dyscalculia” OR “dyscalculias” OR “acalculia” OR “acalculias” OR “reading disorder” OR “reading disorders” OR “reading disability” OR “reading disabilities” OR “mathematics disorder” OR “mathematics disorders” OR “disorder of written expression” OR “written expression disorder” OR “written expression disorders” OR “sensory processing disorder” OR “sensory processing disorders” OR “auditory processing disorder” OR “auditory processing disorders” OR “visual processing disorder” OR “visual processing disorders” OR “dyspraxia” OR “dyspraxias” OR “developmental coordination disorder”)**) AND (**MH "Child+" OR MH "Adolescence+" OR MH "Pediatrics+" OR MH "Pediatric Nursing+" OR MH "Pediatric Nurses+" OR MH "Pediatric Nurse Practitioners+" OR MH "Hospitals, Pediatric" OR MH "Pediatric Units+" OR MH "Pediatric Emergency Nursing" OR MH "Pediatric Anesthesia" OR MH "Adolescent Medicine" OR MH "Adolescent, Hospitalized" OR MH "Adolescent Health Services" OR MH "Child Health Services+" OR TI(“child” OR “children” OR “infant” OR “infants” OR “baby” OR “babies” OR “toddler” OR “toddlers” OR “adolescent” OR “adolescents” OR “adolescence” OR “youth” OR “youths” OR “teen” OR “teens” OR “teenager” OR “teenagers” OR “young person” OR “young people” OR “pediatric” OR “pediatrics” OR “paediatric” OR “paediatrics”) OR AB(“child” OR “children” OR “infant” OR “infants” OR “baby” OR “babies” OR “toddler” OR “toddlers” OR “adolescent” OR “adolescents” OR “adolescence” OR “youth” OR “youths” OR “teen” OR “teens” OR “teenager” OR “teenagers” OR “young person” OR “young people” OR “pediatric” OR “pediatrics” OR “paediatric” OR “paediatrics”)**) AND (**TI(“procedural” OR “procedure” OR “procedures” OR “imaging” OR “radiological” OR “radiology” OR “surgical” OR “needle” OR “needles” OR “venipuncture” OR “venepuncture” OR “healthcare encounters” OR “healthcare encounter” OR “hospital stay” OR “hospital stays” OR “hospital visit” OR “hospital visits” OR “hospitalisation” OR “hospitalisations” OR “hospitalization” OR “hospitalizations” OR “hospitalised” OR “hospitalized” OR “presentations” OR “presentations” OR “pain”) OR AB(“procedural” OR “procedure” OR “procedures” OR “imaging” OR “radiological” OR “radiology” OR “surgical” OR “needle” OR “needles” OR “venipuncture” OR “venepuncture” OR “healthcare encounters” OR “healthcare encounter” OR “hospital stay” OR “hospital stays” OR “hospital visit” OR “hospital visits”)**) AND (**(TI(“support” OR “supportive” OR “care” OR “adjustments” OR “adjustment” OR “accommodation” OR “accommodations” OR “special considerations” OR “special needs” OR “additional needs” OR “individual needs”) OR AB(“support” OR “supportive” OR “care” OR “adjustments” OR “adjustment” OR “accommodation” OR “accommodations” OR “special considerations” OR “special needs” OR “additional needs” OR “individual needs”)) **OR (**(TI(“intervention” OR “interventions” OR “management” OR “procedural” OR “strategies” OR “strategy” OR “technique” OR “techniques” OR “approach” OR “approaches” OR “protocol” OR “clinical pathway” OR “undergoing”) OR AB(“intervention” OR “interventions” OR “management” OR “procedural” OR “strategies” OR “strategy” OR “technique” OR “techniques” OR “approach” OR “approaches” OR “protocol” OR “clinical pathway” OR “undergoing”)) **AND** (TI(“behaviour” OR “behavioural” OR “psychological” OR “hypnosis” OR “distraction” OR “diversion” OR “diversional” OR “pharmacological” OR “sedation” OR “sedative” OR “sedatives” OR “sedate” OR “sedates” OR “sedated” OR “sedating” OR “analgesic” OR “analgesics” OR “anaesthetic” OR “anaesthetics” OR “anesthetic” OR “anesthetics” OR “anaesthesia” OR “anesthesia” OR “physical” OR “heat” OR “cold” OR “buzzy” OR “vibration” OR “restraint” OR “restraints” OR “child-focused” OR “child focused” OR “child-centred” OR “child centred” OR “child-centered” OR “child centered”) OR AB(“behaviour” OR “behavioural” OR “psychological” OR “hypnosis” OR “distraction” OR “diversion” OR “diversional” OR “pharmacological” OR “sedation” OR “sedative” OR “sedatives” OR “sedate” OR “sedates” OR “sedated” OR “sedating” OR “analgesic” OR “analgesics” OR “anaesthetic” OR “anaesthetics” OR “anesthetic” OR “anesthetics” OR “anaesthesia” OR “anesthesia” OR “physical” OR “heat” OR “cold” OR “buzzy” OR “vibration” OR “restraint” OR “restraints” OR “child-focused” OR “child focused” OR “child-centred” OR “child centred” OR “child-centered” OR “child centered”))**)) AND (**LA English**) AND** PY 2014-2025 **NOT (**(MH "Animals+" OR MH "Animal Studies" OR TI animal model*) NOT MH "Human"**)**

**APA PsycInfo (EBSCOhost)** 1302 results

Includes APA Thesaurus of Psychological Index Terms

**(**DE "Neurodevelopmental Disorders" OR DE "Attention Deficit Disorder" OR DE "Autism Spectrum Disorders" OR DE "Attention Deficit Disorder with Hyperactivity" OR DE "Dyspraxia" OR DE "Intellectual Development Disorder" OR DE "Learning Disorders" OR DE "Social Communication Disorder" OR DE "Speech Sound Disorder" OR DE "Stereotypic Movement Disorder" OR DE "Stuttering" OR DE "Tic Disorders" OR DE "Tourette Syndrome" OR DE "Neurodiversity" OR DE "Communication Disorders" OR DE "Hearing Disorders" OR DE "Language Disorders" OR DE "Social Communication Disorder" OR TI(“neurodevelopmental” OR “neurodivergent” OR “neurodiversity” OR “neurodiverse” OR “autism” OR “autistic” OR “Asperger’s” OR “Aspergers” OR “Asperger” OR “Aspergic” OR “ASD” OR “attention deficit hyperactivity disorder” OR “attention deficit hyperactivity disorders” OR “attention-deficit/hyperactivity disorder” OR “attention-deficit/hyperactivity disorders” OR “attention deficit-hyperactivity disorder” OR “attention deficit-hyperactivity disorders” OR “ADHD” OR “ADDH” OR “attention deficit disorder” OR “attention deficit disorders” OR “ADD” OR “developmental disorder” OR “developmental disorders” OR “developmental disability” OR “developmental disabilities” OR “childhood disintegrative disorder” OR “intellectual disabilities” OR “intellectual disability” OR “development disorder” OR “development disorders” OR “intellectual disorders” OR “intellectual disorder” OR “communication disorders” OR “communication disorder” OR “communicative disorder” OR “communicative disorders” OR “communication disabilities” OR “communication disability” OR “communicative dysfunction” OR “communicative dysfunctions” OR “language disorders” OR “language disorder” OR “language impairment” OR “language impairments” OR “learning disorder” OR “learning disorders” OR “learning disability” OR “learning disabilities” OR “learning disturbance” OR “learning disturbances” OR “speech sound disorder” OR “speech sound disorders” OR “childhood-onset fluency disorder” OR “childhood-onset fluency disorders” OR “childhood onset fluency disorders” OR “childhood onset fluency disorders” OR “stuttering” OR “phonological disorder” OR “phonological disorders” OR “mental retardation” OR “developmental coordination disorder” OR “developmental coordination disorders” OR “stereotypic movement disorder” OR “stereotypic movement disorders” OR “tic disorder” OR “tic disorders” OR “Tourette’s” OR “Tourette” OR “Tourettes” OR “dyslexia” OR “dyslexias” OR “dyslexic” OR “dyslexics” OR “dysgraphia” OR “dysgraphias” OR “agraphia” OR “agraphias” OR “dyscalculia” OR “dyscalculias” OR “acalculia” OR “acalculias” OR “reading disorder” OR “reading disorders” OR “reading disability” OR “reading disabilities” OR “mathematics disorder” OR “mathematics disorders” OR “disorder of written expression” OR “written expression disorder” OR “written expression disorders” OR “sensory processing disorder” OR “sensory processing disorders” OR “auditory processing disorder” OR “auditory processing disorders” OR “visual processing disorder” OR “visual processing disorders” OR “dyspraxia” OR “dyspraxias” OR “developmental coordination disorder”) OR AB(“neurodevelopmental” OR “neurodivergent” OR “neurodiversity” OR “neurodiverse” OR “autism” OR “autistic” OR “Asperger’s” OR “Aspergers” OR “Asperger” OR “Aspergic” OR “ASD” OR “attention deficit hyperactivity disorder” OR “attention deficit hyperactivity disorders” OR “attention-deficit/hyperactivity disorder” OR “attention-deficit/hyperactivity disorders” OR “attention deficit-hyperactivity disorder” OR “attention deficit-hyperactivity disorders” OR “ADHD” OR “ADDH” OR “attention deficit disorder” OR “attention deficit disorders” OR “ADD” OR “developmental disorder” OR “developmental disorders” OR “developmental disability” OR “developmental disabilities” OR “childhood disintegrative disorder” OR “intellectual disabilities” OR “intellectual disability” OR “development disorder” OR “development disorders” OR “intellectual disorders” OR “intellectual disorder” OR “communication disorders” OR “communication disorder” OR “communicative disorder” OR “communicative disorders” OR “communication disabilities” OR “communication disability” OR “communicative dysfunction” OR “communicative dysfunctions” OR “language disorders” OR “language disorder” OR “language impairment” OR “language impairments” OR “learning disorder” OR “learning disorders” OR “learning disability” OR “learning disabilities” OR “learning disturbance” OR “learning disturbances” OR “speech sound disorder” OR “speech sound disorders” OR “childhood-onset fluency disorder” OR “childhood-onset fluency disorders” OR “childhood onset fluency disorders” OR “childhood onset fluency disorders” OR “stuttering” OR “phonological disorder” OR “phonological disorders” OR “mental retardation” OR “developmental coordination disorder” OR “developmental coordination disorders” OR “stereotypic movement disorder” OR “stereotypic movement disorders” OR “tic disorder” OR “tic disorders” OR “Tourette’s” OR “Tourette” OR “Tourettes” OR “dyslexia” OR “dyslexias” OR “dyslexic” OR “dyslexics” OR “dysgraphia” OR “dysgraphias” OR “agraphia” OR “agraphias” OR “dyscalculia” OR “dyscalculias” OR “acalculia” OR “acalculias” OR “reading disorder” OR “reading disorders” OR “reading disability” OR “reading disabilities” OR “mathematics disorder” OR “mathematics disorders” OR “disorder of written expression” OR “written expression disorder” OR “written expression disorders” OR “sensory processing disorder” OR “sensory processing disorders” OR “auditory processing disorder” OR “auditory processing disorders” OR “visual processing disorder” OR “visual processing disorders” OR “dyspraxia” OR “dyspraxias” OR “developmental coordination disorder”)**) AND (**DE "Pediatrics" OR TI(“child” OR “children” OR “infant” OR “infants” OR “baby” OR “babies” OR “toddler” OR “toddlers” OR “adolescent” OR “adolescents” OR “adolescence” OR “youth” OR “youths” OR “teen” OR “teens” OR “teenager” OR “teenagers” OR “young person” OR “young people” OR “pediatric” OR “pediatrics” OR “paediatric” OR “paediatrics”) OR AB(“child” OR “children” OR “infant” OR “infants” OR “baby” OR “babies” OR “toddler” OR “toddlers” OR “adolescent” OR “adolescents” OR “adolescence” OR “youth” OR “youths” OR “teen” OR “teens” OR “teenager” OR “teenagers” OR “young person” OR “young people” OR “pediatric” OR “pediatrics” OR “paediatric” OR “paediatrics”)**) AND (**TI(“procedural” OR “procedure” OR “procedures” OR “imaging” OR “radiological” OR “radiology” OR “surgical” OR “needle” OR “needles” OR “venipuncture” OR “venepuncture” OR “healthcare encounters” OR “healthcare encounter” OR “hospital stay” OR “hospital stays” OR “hospital visit” OR “hospital visits” OR “hospitalisation” OR “hospitalisations” OR “hospitalization” OR “hospitalizations” OR “hospitalised” OR “hospitalized” OR “presentations” OR “presentations” OR “pain”) OR AB(“procedural” OR “procedure” OR “procedures” OR “imaging” OR “radiological” OR “radiology” OR “surgical” OR “needle” OR “needles” OR “venipuncture” OR “venepuncture” OR “healthcare encounters” OR “healthcare encounter” OR “hospital stay” OR “hospital stays” OR “hospital visit” OR “hospital visits”)**) AND (**(TI(“support” OR “supportive” OR “care” OR “adjustments” OR “adjustment” OR “accommodation” OR “accommodations” OR “special considerations” OR “special needs” OR “additional needs” OR “individual needs”) OR AB(“support” OR “supportive” OR “care” OR “adjustments” OR “adjustment” OR “accommodation” OR “accommodations” OR “special considerations” OR “special needs” OR “additional needs” OR “individual needs”)) **OR (**(TI(“intervention” OR “interventions” OR “management” OR “procedural” OR “strategies” OR “strategy” OR “technique” OR “techniques” OR “approach” OR “approaches” OR “protocol” OR “clinical pathway” OR “undergoing”) OR AB(“intervention” OR “interventions” OR “management” OR “procedural” OR “strategies” OR “strategy” OR “technique” OR “techniques” OR “approach” OR “approaches” OR “protocol” OR “clinical pathway” OR “undergoing”)) **AND** (TI(“behaviour” OR “behavioural” OR “psychological” OR “hypnosis” OR “distraction” OR “diversion” OR “diversional” OR “pharmacological” OR “sedation” OR “sedative” OR “sedatives” OR “sedate” OR “sedates” OR “sedated” OR “sedating” OR “analgesic” OR “analgesics” OR “anaesthetic” OR “anaesthetics” OR “anesthetic” OR “anesthetics” OR “anaesthesia” OR “anesthesia” OR “physical” OR “heat” OR “cold” OR “buzzy” OR “vibration” OR “restraint” OR “restraints” OR “child-focused” OR “child focused” OR “child-centred” OR “child centred” OR “child-centered” OR “child centered”) OR AB(“behaviour” OR “behavioural” OR “psychological” OR “hypnosis” OR “distraction” OR “diversion” OR “diversional” OR “pharmacological” OR “sedation” OR “sedative” OR “sedatives” OR “sedate” OR “sedates” OR “sedated” OR “sedating” OR “analgesic” OR “analgesics” OR “anaesthetic” OR “anaesthetics” OR “anesthetic” OR “anesthetics” OR “anaesthesia” OR “anesthesia” OR “physical” OR “heat” OR “cold” OR “buzzy” OR “vibration” OR “restraint” OR “restraints” OR “child-focused” OR “child focused” OR “child-centred” OR “child centred” OR “child-centered” OR “child centered”))**)) AND (**LA English**) AND** PY 2014-2025

**Cochrane Library (Wiley)** 1042 results (90 Cochrane Reviews and 952 CENTRAL Trials)

Includes MeSH

Advanced search > Search manager

Further limited CENTRAL Trials tab results to “Year first published” from 2014.

Link to saved search - <https://www.cochranelibrary.com/advanced-search/search-manager?search=7622128>

ID Search Hits

#1 MeSH descriptor: [Neurodevelopmental Disorders] explode all trees 11538

#2 MeSH descriptor: [Communication Disorders] explode all trees 2612

#3 MeSH descriptor: [Intellectual Disability] explode all trees 2099

#4 (“neurodevelopmental” OR “neurodivergent” OR “neurodiversity” OR “neurodiverse” OR “autism” OR “autistic” OR “Asperger’s” OR “Aspergers” OR “Asperger” OR “Aspergic” OR “ASD” OR “attention deficit hyperactivity disorder” OR “attention deficit hyperactivity disorders” OR “ADHD” OR “ADDH” OR “attention deficit disorder” OR “attention deficit disorders” OR “ADD” OR “developmental disorder” OR “developmental disorders” OR “developmental disability” OR “developmental disabilities” OR “childhood disintegrative disorder” OR “intellectual disabilities” OR “intellectual disability” OR “development disorder” OR “development disorders” OR “intellectual disorders” OR “intellectual disorder” OR “communication disorders” OR “communication disorder” OR “communicative disorder” OR “communicative disorders” OR “communication disabilities” OR “communication disability” OR “communicative dysfunction” OR “communicative dysfunctions” OR “language disorders” OR “language disorder” OR “language impairment” OR “language impairments” OR “learning disorder” OR “learning disorders” OR “learning disability” OR “learning disabilities” OR “learning disturbance” OR “learning disturbances” OR “speech sound disorder” OR “speech sound disorders” OR “childhood onset fluency disorders” OR “childhood onset fluency disorders” OR “stuttering” OR “phonological disorder” OR “phonological disorders” OR “mental retardation” OR “developmental coordination disorder” OR “developmental coordination disorders” OR “stereotypic movement disorder” OR “stereotypic movement disorders” OR “tic disorder” OR “tic disorders” OR “Tourette’s” OR “Tourette” OR “Tourettes” OR “dyslexia” OR “dyslexias” OR “dyslexic” OR “dyslexics” OR “dysgraphia” OR “dysgraphias” OR “agraphia” OR “agraphias” OR “dyscalculia” OR “dyscalculias” OR “acalculia” OR “acalculias” OR “reading disorder” OR “reading disorders” OR “reading disability” OR “reading disabilities” OR “mathematics disorder” OR “mathematics disorders” OR “disorder of written expression” OR “written expression disorder” OR “written expression disorders” OR “sensory processing disorder” OR “sensory processing disorders” OR “auditory processing disorder” OR “auditory processing disorders” OR “visual processing disorder” OR “visual processing disorders” OR “dyspraxia” OR “dyspraxias” OR “developmental coordination disorder”):ti,ab 39117

#5 #1 OR #2 OR #3 OR #4 43959

#6 MeSH descriptor: [Child] explode all trees 83857

#7 MeSH descriptor: [Infant] explode all trees 47086

#8 MeSH descriptor: [Adolescent] explode all trees 139606

#9 MeSH descriptor: [Pediatrics] explode all trees 1060

#10 MeSH descriptor: [Pediatric Nursing] explode all trees 304

#11 MeSH descriptor: [Nurses, Pediatric] explode all trees 9

#12 MeSH descriptor: [Pediatric Nurse Practitioners] explode all trees 3

#13 MeSH descriptor: [Hospitals, Pediatric] explode all trees 297

#14 MeSH descriptor: [Pediatric Emergency Medicine] explode all trees 15

#15 MeSH descriptor: [Pediatric Anesthesia] explode all trees 3

#16 MeSH descriptor: [Adolescent Medicine] explode all trees 9

#17 MeSH descriptor: [Adolescent, Hospitalized] explode all trees 10

#18 MeSH descriptor: [Adolescent Health Services] explode all trees 231

#19 MeSH descriptor: [Child Health Services] explode all trees 1294

#20 (“child” OR “children” OR “infant” OR “infants” OR “baby” OR “babies” OR “toddler” OR “toddlers” OR “adolescent” OR “adolescents” OR “adolescence” OR “youth” OR “youths” OR “teen” OR “teens” OR “teenager” OR “teenagers” OR “young person” OR “young people” OR “pediatric” OR “pediatrics” OR “paediatric” OR “paediatrics”):ti,ab 238533

#21 #6 OR #7 OR #8 OR #9 OR #10 OR #11 OR #12 OR #13 OR #14 OR #15 OR #16 OR #17 OR #18 OR #19 OR #20 351512

#22 (“procedural” OR “procedure” OR “procedures” OR “imaging” OR “radiological” OR “radiology” OR “surgical” OR “needle” OR “needles” OR “venipuncture” OR “venepuncture” OR “healthcare encounters” OR “healthcare encounter” OR “hospital stay” OR “hospital stays” OR “hospital visit” OR “hospital visits”):ti,ab 313766

#23 (“hospitalisation” OR “hospitalisations” OR “hospitalization” OR “hospitalizations” OR “hospitalised” OR “hospitalized” OR “presentations” OR “presentations” OR “pain”):ti 89741

#24 #22 OR #23 383038

#25 (((“support” OR “supportive” OR “care” OR “adjustments” OR “adjustment” OR “accommodation” OR “accommodations” OR “special considerations” OR “special needs” OR “additional needs” OR “individual needs”) OR ((“intervention” OR “interventions” OR “management” OR “procedural” OR “strategies” OR “strategy” OR “technique” OR “techniques” OR “approach” OR “approaches” OR “protocol” OR “clinical pathway” OR “undergoing”) AND (“behaviour” OR “behavioural” OR “psychological” OR “hypnosis” OR “distraction” OR “diversion” OR “diversional” OR “pharmacological” OR “sedation” OR “sedative” OR “sedatives” OR “sedate” OR “sedates” OR “sedated” OR “sedating” OR “analgesic” OR “analgesics” OR “anaesthetic” OR “anaesthetics” OR “anesthetic” OR “anesthetics” OR “anaesthesia” OR “anesthesia” OR “physical” OR “heat” OR “cold” OR “buzzy” OR “vibration” OR “restraint” OR “restraints” OR “child-focused” OR “child focused” OR “child-centred” OR “child centred” OR “child-centered” OR “child centered”)))):ti,ab 578780

#26 #5 AND #21 AND #24 AND #25 with Cochrane Library publication date from Jan 2014 to present 1208

**Web of Science (Clarivate)** 2639 results

Web of Science Core Collection, All Editions

Advanced search > Query preview

Refined results to relevant publication years and English language

**(**TI=(“neurodevelopmental” OR “neurodivergent” OR “neurodiversity” OR “neurodiverse” OR “autism” OR “autistic” OR “Asperger’s” OR “Aspergers” OR “Asperger” OR “Aspergic” OR “ASD” OR “attention deficit hyperactivity disorder” OR “attention deficit hyperactivity disorders” OR “attention-deficit/hyperactivity disorder” OR “attention-deficit/hyperactivity disorders” OR “attention deficit-hyperactivity disorder” OR “attention deficit-hyperactivity disorders” OR “ADHD” OR “ADDH” OR “attention deficit disorder” OR “attention deficit disorders” OR “ADD” OR “developmental disorder” OR “developmental disorders” OR “developmental disability” OR “developmental disabilities” OR “childhood disintegrative disorder” OR “intellectual disabilities” OR “intellectual disability” OR “development disorder” OR “development disorders” OR “intellectual disorders” OR “intellectual disorder” OR “communication disorders” OR “communication disorder” OR “communicative disorder” OR “communicative disorders” OR “communication disabilities” OR “communication disability” OR “communicative dysfunction” OR “communicative dysfunctions” OR “language disorders” OR “language disorder” OR “language impairment” OR “language impairments” OR “learning disorder” OR “learning disorders” OR “learning disability” OR “learning disabilities” OR “learning disturbance” OR “learning disturbances” OR “speech sound disorder” OR “speech sound disorders” OR “childhood-onset fluency disorder” OR “childhood-onset fluency disorders” OR “childhood onset fluency disorders” OR “childhood onset fluency disorders” OR “stuttering” OR “phonological disorder” OR “phonological disorders” OR “mental retardation” OR “developmental coordination disorder” OR “developmental coordination disorders” OR “stereotypic movement disorder” OR “stereotypic movement disorders” OR “tic disorder” OR “tic disorders” OR “Tourette’s” OR “Tourette” OR “Tourettes” OR “dyslexia” OR “dyslexias” OR “dyslexic” OR “dyslexics” OR “dysgraphia” OR “dysgraphias” OR “agraphia” OR “agraphias” OR “dyscalculia” OR “dyscalculias” OR “acalculia” OR “acalculias” OR “reading disorder” OR “reading disorders” OR “reading disability” OR “reading disabilities” OR “mathematics disorder” OR “mathematics disorders” OR “disorder of written expression” OR “written expression disorder” OR “written expression disorders” OR “sensory processing disorder” OR “sensory processing disorders” OR “auditory processing disorder” OR “auditory processing disorders” OR “visual processing disorder” OR “visual processing disorders” OR “dyspraxia” OR “dyspraxias” OR “developmental coordination disorder”) OR AB=(“neurodevelopmental” OR “neurodivergent” OR “neurodiversity” OR “neurodiverse” OR “autism” OR “autistic” OR “Asperger’s” OR “Aspergers” OR “Asperger” OR “Aspergic” OR “ASD” OR “attention deficit hyperactivity disorder” OR “attention deficit hyperactivity disorders” OR “attention-deficit/hyperactivity disorder” OR “attention-deficit/hyperactivity disorders” OR “attention deficit-hyperactivity disorder” OR “attention deficit-hyperactivity disorders” OR “ADHD” OR “ADDH” OR “attention deficit disorder” OR “attention deficit disorders” OR “ADD” OR “developmental disorder” OR “developmental disorders” OR “developmental disability” OR “developmental disabilities” OR “childhood disintegrative disorder” OR “intellectual disabilities” OR “intellectual disability” OR “development disorder” OR “development disorders” OR “intellectual disorders” OR “intellectual disorder” OR “communication disorders” OR “communication disorder” OR “communicative disorder” OR “communicative disorders” OR “communication disabilities” OR “communication disability” OR “communicative dysfunction” OR “communicative dysfunctions” OR “language disorders” OR “language disorder” OR “language impairment” OR “language impairments” OR “learning disorder” OR “learning disorders” OR “learning disability” OR “learning disabilities” OR “learning disturbance” OR “learning disturbances” OR “speech sound disorder” OR “speech sound disorders” OR “childhood-onset fluency disorder” OR “childhood-onset fluency disorders” OR “childhood onset fluency disorders” OR “childhood onset fluency disorders” OR “stuttering” OR “phonological disorder” OR “phonological disorders” OR “mental retardation” OR “developmental coordination disorder” OR “developmental coordination disorders” OR “stereotypic movement disorder” OR “stereotypic movement disorders” OR “tic disorder” OR “tic disorders” OR “Tourette’s” OR “Tourette” OR “Tourettes” OR “dyslexia” OR “dyslexias” OR “dyslexic” OR “dyslexics” OR “dysgraphia” OR “dysgraphias” OR “agraphia” OR “agraphias” OR “dyscalculia” OR “dyscalculias” OR “acalculia” OR “acalculias” OR “reading disorder” OR “reading disorders” OR “reading disability” OR “reading disabilities” OR “mathematics disorder” OR “mathematics disorders” OR “disorder of written expression” OR “written expression disorder” OR “written expression disorders” OR “sensory processing disorder” OR “sensory processing disorders” OR “auditory processing disorder” OR “auditory processing disorders” OR “visual processing disorder” OR “visual processing disorders” OR “dyspraxia” OR “dyspraxias” OR “developmental coordination disorder”)**) AND (**TI=(“child” OR “children” OR “infant” OR “infants” OR “baby” OR “babies” OR “toddler” OR “toddlers” OR “adolescent” OR “adolescents” OR “adolescence” OR “youth” OR “youths” OR “teen” OR “teens” OR “teenager” OR “teenagers” OR “young person” OR “young people” OR “pediatric” OR “pediatrics” OR “paediatric” OR “paediatrics”) OR AB=(“child” OR “children” OR “infant” OR “infants” OR “baby” OR “babies” OR “toddler” OR “toddlers” OR “adolescent” OR “adolescents” OR “adolescence” OR “youth” OR “youths” OR “teen” OR “teens” OR “teenager” OR “teenagers” OR “young person” OR “young people” OR “pediatric” OR “pediatrics” OR “paediatric” OR “paediatrics”)**) AND (**TI=(“procedural” OR “procedure” OR “procedures” OR “imaging” OR “radiological” OR “radiology” OR “surgical” OR “needle” OR “needles” OR “venipuncture” OR “venepuncture” OR “healthcare encounters” OR “healthcare encounter” OR “hospital stay” OR “hospital stays” OR “hospital visit” OR “hospital visits” OR “hospitalisation” OR “hospitalisations” OR “hospitalization” OR “hospitalizations” OR “hospitalised” OR “hospitalized” OR “presentations” OR “presentations” OR “pain”) OR AB=(“procedural” OR “procedure” OR “procedures” OR “imaging” OR “radiological” OR “radiology” OR “surgical” OR “needle” OR “needles” OR “venipuncture” OR “venepuncture” OR “healthcare encounters” OR “healthcare encounter” OR “hospital stay” OR “hospital stays” OR “hospital visit” OR “hospital visits”)**) AND (**(TI=(“support” OR “supportive” OR “care” OR “adjustments” OR “adjustment” OR “accommodation” OR “accommodations” OR “special considerations” OR “special needs” OR “additional needs” OR “individual needs”) OR AB=(“support” OR “supportive” OR “care” OR “adjustments” OR “adjustment” OR “accommodation” OR “accommodations” OR “special considerations” OR “special needs” OR “additional needs” OR “individual needs”)) **OR (**(TI=(“intervention” OR “interventions” OR “management” OR “procedural” OR “strategies” OR “strategy” OR “technique” OR “techniques” OR “approach” OR “approaches” OR “protocol” OR “clinical pathway” OR “undergoing”) OR AB=(“intervention” OR “interventions” OR “management” OR “procedural” OR “strategies” OR “strategy” OR “technique” OR “techniques” OR “approach” OR “approaches” OR “protocol” OR “clinical pathway” OR “undergoing”)) **AND** (TI=(“behaviour” OR “behavioural” OR “psychological” OR “hypnosis” OR “distraction” OR “diversion” OR “diversional” OR “pharmacological” OR “sedation” OR “sedative” OR “sedatives” OR “sedate” OR “sedates” OR “sedated” OR “sedating” OR “analgesic” OR “analgesics” OR “anaesthetic” OR “anaesthetics” OR “anesthetic” OR “anesthetics” OR “anaesthesia” OR “anesthesia” OR “physical” OR “heat” OR “cold” OR “buzzy” OR “vibration” OR “restraint” OR “restraints” OR “child-focused” OR “child focused” OR “child-centred” OR “child centred” OR “child-centered” OR “child centered”) OR AB=(“behaviour” OR “behavioural” OR “psychological” OR “hypnosis” OR “distraction” OR “diversion” OR “diversional” OR “pharmacological” OR “sedation” OR “sedative” OR “sedatives” OR “sedate” OR “sedates” OR “sedated” OR “sedating” OR “analgesic” OR “analgesics” OR “anaesthetic” OR “anaesthetics” OR “anesthetic” OR “anesthetics” OR “anaesthesia” OR “anesthesia” OR “physical” OR “heat” OR “cold” OR “buzzy” OR “vibration” OR “restraint” OR “restraints” OR “child-focused” OR “child focused” OR “child-centred” OR “child centred” OR “child-centered” OR “child centered”))**))**

**Supplementary Table 1: Data extraction table**

| Category | Sub-Category | Description of Data to be Extracted |
| --- | --- | --- |
| Study characteristics | Author | Name of first author |
|  | Year | Publication year |
|  | Country | Country where study was conducted |
|  | Study Design | Study Design (e.g. RCT, cohort study etc.) |
|  | Study setting | Hospital (inpatient)  Hospital (outpatient)  Community health centre |
|  | Speciality | Oncology/Haematology  Medical/Surgical  Perioperative  Critical Care (ICU)  Emergency Department  Dental  Psychiatry  Other |
|  | Funding Source | Government  Philanthropy  Hospital  University  Private  None  Not stated  Other |
| Participant Characteristics | Number of Children | Number |
|  | Number of Parents/Caregivers | Number |
|  | Number of Clinicians | Number |
|  | Age range | Age distribution of participants |
|  | Neurodivergent diagnosis (DSM-VTR) | Autism Spectrum Disorder (ASD)  Attention Deficit Hyperactivity Disorder (ADHD) including ADD  Communication disorders  Intellectual Disorders  Neurodevelopmental motor disorders (includes cerebral palsy, Down’s syndrome)  Specific Learning Disorders (such as dyslexia)  Other |
|  | Comorbidities | Additional diagnoses or conditions |
|  | Communication Level | Verbal  non-verbal  assisted communication  Not stated |
|  | Type of Procedure | Venipuncture  Vaccination  Dressing change  Radiological procedures  Diagnostic tests  Emergency procedure  Dental  Other |
|  | Duration of procedure | Length of procedure |
|  | Setting | Where procedure was performed  Bedside  Theatre  Procedure room  Dental clinic  Other |
|  | Support Person Present | Yes  No  Not stated |
| Intervention Details | Type of Support Strategy | Patient care plan or pathway  Hospital-wide strategy  Visit preparation and support  Play-based  Technology (e.g. VR)  Pharmacological agents  Physical Restraints  Other |
|  | Type of Support Strategy | Description of intervention/support provided (free text) |
|  | Phamacological agents addressed for: | Pain  Anxiety  Sedation  Other |
|  |  |  |
|  | Timing of Implementation | Pre-procedure  During procedure  Post-procedure  Not applicable  Other |
|  | Provider of Support | Doctor  Nurses  Occupational Therapist  Parents/Caregiver  Dentist  Dental Assistants  Not applicable  Other |
|  | Additional information | Free text |
| Assessment Methods | Pain assessment by: | Child  Parent  Observer (Clinician/Researcher)  Not appliable/Not used |
|  | Pain Assessment Tool Assessment | Tools/scales used to measure pain  Face, Legs, Activity, Cry, Consolability (FLACC) Scale  Wong-Baker FACES Pain Scale  Numeric Rating Scale (NRS)  Non-Communicating Children's Pain Checklist  Not used  Unsure  Other |
|  | Distress assessed by: | Child  Parent  Observer (Clinician/Researcher)  Not appliable/Not used |
|  | Distress Assessment Tool | Tools/scales used to measure distress  Observational Scale of Behavioral Distress (OSBD)  Children's Fear Scale  State-Trait Anxiety Inventory (STAI)  Physiological measures  Not used  Unsure  Other |
|  | Physiological Measures | Any physiological measurements taken (free text) |
|  | Parent/Caregiver Report | Input from family members (free text) |
|  | Observer (Clinician/Researcher) Report | Clinical staff observations (free text) |
|  | Timing of Assessment | When assessments were conducted  Pre-procedure  During procedure  Post-procedure  Not applicable  Other |
| Outcomes | Survey/Descriptive design - summary of results | Free text |
|  | Pain Scores | Free text |
|  | Distress Levels | Free text |
|  | Global judgement of satisfaction with procedural support | Positive  Mixed  No effect  Other |
|  | Adverse Events | Any negative outcomes reported (free text) |
|  | Follow-up Outcomes | Free text |
|  | Emotional response | Free text |
|  | Adverse Events | Free text |
|  | Additional information | Free text |
| Implementation Factors | Barriers Identified | Free text |
|  | Facilitators | Free text |
|  | Resource Requirements | Free text |
|  | Cost Implications | Free text |
|  | Feasibility Issues | Free text |
|  | Sustainability | Free text |
| Key Findings |  | Primary outcomes and results (free text) |
| Limitations |  | Study limitations identified (free text) |
| Future Research Needs |  | Authors' suggestions for practice Identified research gaps (free text) |

**Supplementary Material 2: References of included studies (N=144)**

1. Aarts, L. A. M., van Geffen, G. J., Smedema, E. A. L., & Smits, R. M. (2023). Therapeutic communication improves patient comfort during venipuncture in children: a single-blinded intervention study. *Eur J Pediatr*, *182*(9), 3871-3881. https://doi.org/10.1007/s00431-023-05036-7
2. Abdelrahman, M. A., Alhebsi, M. A., Almulla, S. I., Rahimi, E. A., Badouh, H., Almehairbi, A., Ghasem, F., & Rawashdeh, M. (2024). Exploration of radiographers' knowledge, attitudes, and practices in delivering healthcare to children with autism spectrum disorder. *Radiography (Lond)*, *30*(1), 116-122. https://doi.org/10.1016/j.radi.2023.10.017
3. Abulebda, K., Louer, R., Lutfi, R., & Ahmed, S. S. (2018). A Comparison of Safety and Efficacy of Dexmedetomidine and Propofol in Children with Autism and Autism Spectrum Disorders Undergoing Magnetic Resonance Imaging. *J Autism Dev Disord*, *48*(9), 3127-3132. https://doi.org/10.1007/s10803-018-3582-1
4. Ahmed, S. S., Unland, T., Slaven, J. E., Nitu, M. E., & Rigby, M. R. (2014). Successful use of intravenous dexmedetomidine for magnetic resonance imaging sedation in autistic children. *South Med J*, *107*(9), 559-564. https://doi.org/10.14423/SMJ.0000000000000160
5. Ahuja, R., Jyoti, B., Shewale, V., Shetty, S., Subudhi, S. K., & Kaur, M. (2016). Comparative Evaluation of Pediatric Patients with Mental Retardation undergoing Dental Treatment under General Anesthesia: A Retrospective Analysis. *J Contemp Dent Pract*, *17*(8), 675-678. https://doi.org/10.5005/jp-journals-10024-1911
6. Alfarraj, J., Louise Gray, J., Alargan, T., Alkathiri, M., Alshehri, R., & Almarwan, M. (2024). Dental treatment provided to special needs children under general anesthesia in a tertiary care hospital - A cross sectional retrospective study. *Saudi Dent J*, *36*(4), 579-583. https://doi.org/10.1016/j.sdentj.2024.01.003
7. Alghafis, B., Alharbi, A., Al-Haj Ali, S., Alsineedi, F., & Alsudairi, O. (2023). Dental Treatment Characteristics of Autistic Children and Differences in Dental Procedures under General Anesthesia Relative to Healthy Counterparts. *Children (Basel)*, *10*(3). https://doi.org/10.3390/children10030466
8. AlHumaid, J., Tesini, D., Finkelman, M., & Loo, C. Y. (2016). Effectiveness of the D-TERMINED Program of Repetitive Tasking for Children with Autism Spectrum Disorder. *J Dent Child (Chic)*, *83*(1), 16-21. https://www.ingentaconnect.com/content/aapd/jodc/2016/00000083/00000001/art00003;jsessionid=3tin60r5jdt4d.x-ic-live-02
9. Al-Jabri, B., Alnuwaiser, S., Abdulghaffar, H., Almuhanna, R., Salaam, S., Brika, R., Addas, A., & Bedaiwi, H. (2023). Healthcare Experience of Pediatric Patients with Autism Spectrum Disorders in Saudi Arabia: A Cross-Sectional Study. *Pediatr Rep*, *15*(3), 452-461. https://doi.org/10.3390/pediatric15030042
10. Aljubour, A. A., AbdElBaki, M., El Meligy, O., Al Jabri, B., & Sabbagh, H. (2024). Culturally Adapted Dental Visual Aids Effect on Behavior Management during Dental Visits in Children with Autism Spectrum Disorder. *J Contemp Dent Pract*, *25*(1), 20-28. https://doi.org/10.5005/jp-journals-10024-3620
11. Alvares, G. A., Mekertichian, K., Rose, F., Vidler, S., & Whitehouse, A. J. O. (2023). Dental care experiences and clinical phenotypes in children on the autism spectrum. *Spec Care Dentist*, *43*(1), 17-28. https://doi.org/10.1111/scd.12746
12. Anderson, K. L., Self, T. L., & Carlson, B. N. (2017). Interprofessional Collaboration of Dental Hygiene and Communication Sciences & Disorders Students to Meet Oral Health Needs of Children with Autism. *J Allied Health*, *46*(4), e97-e101.
13. Ben-Pazi, H., Cohen, A., Kroyzer, N., Lotem-Ophir, R., Shvili, Y., Winter, G., Deutsch, L., & Pollak, Y. (2017). Clown-care reduces pain in children with cerebral palsy undergoing recurrent botulinum toxin injections- A quasi-randomized controlled crossover study. *PLoS One*, *12*(4). https://doi.org/10.1371/journal.pone.0175028
14. Berglund, I. G., Björkman, B., Enskär, K., Faresjö, M., & Huus, K. (2017). Management of Children with Autism Spectrum Disorder in the Anesthesia and Radiographic Context. *J Dev Behav Pediatr*, *38*(3), 187-196. https://doi.org/10.1097/dbp.0000000000000432
15. Bevan, S., Harris, K., Maeder-Chieffo, S., Reswebber, E., Lanahan, D., & Souders, M. (2023). Positive Healthcare Encounters for Children With Autism Spectrum Disorder: Accommodations During Surgical Procedures. *J Perianesth Nurs*, *38*(1), 6-11. https://doi.org/10.1016/j.jopan.2022.05.070
16. Birkett, K., Liddle, M., Jones, E., & Paulson, A. (2022). Matching Level of Clinical Support to Patient Risk When Caring for Children With Behavioral Challenges. *Intellect Dev Disabil*, *60*(1), 32-40. https://doi.org/10.1352/1934-9556-60.1.32
17. Björkman, B., Berglund, I. G., Enskär, K., Faresjö, M., & Huus, K. (2017). Peri-radiographic guidelines for children with autism spectrum disorder: A nationwide survey in Sweden. *Child: care, health and development*, *43*(1), 31-36. https://doi.org/10.1111/cch.12427
18. Bondioli, M., Buzzi, M. C., Buzzi, M., Pelagatti, S., & Senette, C. (2018). *ICT to Support Dental Care of Children with Autism: An Exploratory Study* https://link.springer.com/content/pdf/10.1007/978-3-319-92049-8_34.pdf
19. Broder-Fingert, S., Shui, A., Ferrone, C., Iannuzzi, D., Cheng, E. R., Giauque, A., Connors, S., McDougle, C. J., Donelan, K., Neumeyer, A., & Kuhlthau, K. (2016). A Pilot Study of Autism-Specific Care Plans During Hospital Admission. *Pediatrics*, *137*(Supplement_2), S196-S204. https://doi.org/10.1542/peds.2015-2851R
20. Brown, J. J., Gray, J. M., Roback, M. G., Sethuraman, U., Farooqi, A., & Kannikeswaran, N. (2019). Procedural sedation in children with autism spectrum disorders in the emergency department. *Am J Emerg Med*, *37*(8), 1404-1408. https://doi.org/10.1016/j.ajem.2018.10.025
21. Burnham Riosa, P., Randhawa, A., & Muskat, B. (2024). Autism Comes to the Pediatric Hospital: Perspectives of Child Life Specialists. *J Autism Dev Disord*, *54*(1), 312-325. https://doi.org/10.1007/s10803-022-05776-9
22. Cagetti, M. G., Mastroberardino, S., Campus, S., Olivari, B., Faggioli, R., Lenti, C., & Strohmenger, L. (2015). Dental care protocol based on visual supports for children with autism spectrum disorders. *Med Oral Patol Oral Cir Bucal*, *20*(5), e598-604. https://doi.org/10.4317/medoral.20424
23. Cai, J., Habib, D., Bedos, C., & Santos, B. F. D. (2022). Parents' Perceptions Regarding the Effectiveness of Dental Desensitization for Children with Autism Spectrum Disorder. *Pediatr Dent*, *44*(3), 192-197.
24. Carlone, G., Trombetta, A., Amoroso, S., Poropat, F., Barbi, E., & Cozzi, G. (2019). Intramuscular Dexmedetomidine, a Feasible Option for Children With Autism Spectrum Disorders Needing Urgent Procedural Sedation. *Pediatr Emerg Care*, *35*(6), e116-e117. https://doi.org/10.1097/pec.0000000000001776
25. Carter, L., Harper, J. M., & Luiselli, J. K. (2019). Dental Desensitization for Students with Autism Spectrum Disorder through Graduated Exposure, Reinforcement, and Reinforcement-Fading. *Journal of Developmental & Physical Disabilities*, *31*(2), 161-170. https://doi.org/10.1007/s10882-018-9635-8
26. Chow, C., & Choong, C. T. (2016). Ketamine-based procedural sedation and analgesia for botulinum toxin A injections in children with cerebral palsy. *European Journal of Paediatric Neurology*, *20*(2), 319-322. https://doi.org/10.1016/j.ejpn.2015.11.009
27. Cirio, S., Salerno, C., Mbanefo, S., Oberti, L., Paniura, L., Campus, G., & Cagetti, M. G. (2022). Use of Visual Pedagogy to Help Children with ASDs Facing the First Dental Examination: A Randomized Controlled Trial. *Children (Basel)*, *9*(5). https://doi.org/10.3390/children9050729
28. Clark, L. A., Whitt, S., & Lyons, K. (2019). Improving Communication Between Health Care Providers, Families, and Children with Autism Spectrum Disorder: The Linked Program. *J Perianesth Nurs*, *34*(5), 889-899. https://doi.org/10.1016/j.jopan.2018.12.009
29. Dangulavanich, W., Limsomwong, P., Mitrakul, K., Asvanund, Y., & Arunakul, M. (2017). Factors associated with cooperative levels of Autism Spectrum Disorder children during dental treatments. *European journal of paediatric dentistry*, *18*(3), 231-236. https://doi.org/10.23804/ejpd.201718.03.11
30. Davignon, M. N., Friedlaender, E., Cronholm, P. F., Paciotti, B., & Levy, S. E. (2014). Parent and provider perspectives on procedural care for children with autism spectrum disorders. *J Dev Behav Pediatr*, *35*(3), 207-215. https://doi.org/10.1097/dbp.0000000000000036
31. Delafontaine, A., Presedo, A., Mohamed, D., Lopes, D., Wood, C., & Alberti, C. (2017). Equimolar mixture of nitroux oxyde and oxygen during post-operative physiotherapy in patients with cerebral palsy: A randomized, double-blind, placebo-controlled study. *European Journal of Pain (United Kingdom)*, *21*(10), 1657-1667. https://doi.org/10.1002/ejp.1071
32. Di Nuovo, A., Bamforth, J., Conti, D., Sage, K., Ibbotson, R., Clegg, J., Westaway, A., Arnold, K., & Assoc Comp, M. (2020). *An Explorative Study on Robotics for Supporting Children with Autism Spectrum Disorder during Clinical Procedures* https://dl.acm.org/doi/pdf/10.1145/3371382.3378277
33. Duker, L. S., Polido, J., & Cermak, S. (2021). Sensory adapted dental environments to enhance oral care for children with autism spectrum disorder. *Pediatrics*, *147*(3), 779‐781. https://doi.org/10.1542/peds.147.3_MeetingAbstract.779
34. Dutta, K., Saha, S., Reddy, H., Reenayai, N., Shetty, R. Y., Rai, K., & Nair, M. R. (2024). Reducing Fear and Enhancing Aesthetics: Orthodontic Management with Video Self-Modeling in an Adolescent with Autism Spectrum Disorder. *JOURNAL OF HEALTH AND ALLIED SCIENCES NU*. https://doi.org/10.1055/s-0044-1788051
35. Erickson, L. C., Scott-Van Zeeland, A. A., Hamilton, G., Lincoln, A., & Golomb, B. A. (2014). Brief Report: Approaches to <SUP>31</SUP>P-MRS in Awake, Non-Sedated Children With and Without Autism Spectrum Disorder (vol 42, pg 1120, 2012). *Journal of Autism and Developmental Disorders*, *44*(10), 2671-2677. https://doi.org/10.1007/s10803-013-1821-z
36. Fahy, R., Corbett, M., & Keogh, I. (2020). Improving peri-operative psychosocial interventions for children with autism spectrum disorder undergoing ENT procedures. *J Laryngol Otol*, 1-7. https://doi.org/10.1017/s0022215120002029
37. Fakhruddin, K. S., & El Batawi, H. Y. (2017). Effectiveness of audiovisual distraction in behavior modification during dental caries assessment and sealant placement in children with autism spectrum disorder. *Dent Res J (Isfahan)*, *14*(3), 177-182. https://doi.org/10.4103/1735-3327.208768
38. Fakhruddin, K. S., ElBatawi, H., & El-Damanhoury, H. M. (2018). Behavioral management using sequenced treatment paradigm and audiovisual distraction during dental treatment in children with attention deficit/hyperactivity disorder. *Eur J Dent*, *12*(2), 262-268. https://doi.org/10.4103/ejd.ejd_59_18
39. Garg, P., Passarello, L., O'Hea, C., Lai, T., Reid, N., & Farrell, B. (2024). Developing a community-based model of care for venipuncture in children and young adults with an intellectual disability: a retrospective study. *BMJ Paediatr Open*, *8*(1). https://doi.org/10.1136/bmjpo-2024-002644
40. Gowdham, G., Shetty, A. A., Hegde, A., & Suresh, L. R. (2021). Impact of Music Distraction on Dental Anxiety in Children Having Intellectual Disability. *Int J Clin Pediatr Dent*, *14*(1), 170-174. https://doi.org/10.5005/jp-journals-10005-1902
41. Gupta, N., Brown, C., Deneke, J., Maha, J., & Kong, M. (2019). Utilization of a Novel Pathway in a Tertiary Pediatric Hospital to Meet the Sensory Needs of Acutely Ill Pediatric Patients. *Front Pediatr*, *7*, 367. https://doi.org/10.3389/fped.2019.00367
42. Harvey, P. C., Willis, E. P. E., Brown, D. J., Byrne, A. L., Baldwin, A. P. A., Heard, D., & Augutis, W. (2023). Navigating the care of families with a child or children with autistic spectrum disorder. *J Intellect Disabil*, *27*(4), 912-926. https://doi.org/10.1177/17446295221106001
43. Hee, H. I., Raghavan, K., Tan, A., Ng, K. L., Cruz, M., Yu, H. Y., & Ieee. (2017). *Enhancing Perioperative Transfer of Special Needs Children <i>with THE</i> <i>I</i>-<i>MOVE</i>*
44. Hernández, P., Molina, A. I., Lacave, C., Rusu, C., & Toledano-González, A. (2022). PlanTEA: Supporting Planning and Anticipation for Children with ASD Attending Medical Appointments. *APPLIED SCIENCES-BASEL*, *12*(10). https://doi.org/10.3390/app12105237
45. Hidayatullah, T., Agustiani, H., & Setiawan, A. S. (2018). Behavior management-based applied behaviour analysis within dental examination of children with autism spectrum disorder. *Dental Journal*, *51*(2), 71-75. https://doi.org/10.20473/j.djmkg.v51.i2.p71-75
46. Houx, L., Dubois, A., Brochard, S., & Pons, C. (2020). Do clowns attenuate pain and anxiety undergoing botulinum toxin injections in children? *Annals of Physical and Rehabilitation Medicine*, *63*(5), 393-399. https://doi.org/10.1016/j.rehab.2018.12.004
47. Houx, L., Pons, C., Saudreau, H., Dubois, A., Creusat, M., Le Moine, P., Rémy-Néris, O., Ropars, J., LeReste, J. Y., & Brochard, S. (2021). No pain, no gain? Children with cerebral palsy and their experience with physiotherapy. *Annals of Physical and Rehabilitation Medicine*, *64*(3). https://doi.org/10.1016/j.rehab.2020.10.002
48. Ismail, N., Isa, K. A. M., Hamzah, S. H., & Mokhtar, I. W. (2023). A Randomized Cross-over Trial of Behavior Guidance Techniques on Children with Special Needs during Dental Treatment: The Caregivers' Perceived Mannerisms. *J Int Soc Prev Community Dent*, *13*(6), 500-508. https://doi.org/10.4103/jispcd.JISPCD_52_23
49. Isong, I. A., Rao, S. R., Holifield, C., Iannuzzi, D., Hanson, E., Ware, J., & Nelson, L. P. (2014). Addressing dental fear in children with autism spectrum disorders: a randomized controlled pilot study using electronic screen media. *Clin Pediatr (Phila)*, *53*(3), 230-237. https://doi.org/10.1177/0009922813517169
50. Jensen, E. J., Geisthardt, C., & Sarigiani, P. A. (2020). Working with Children with Autism Spectrum Disorder in a Medical Setting: Insights from Certified Child Life Specialists. *Journal of Autism and Developmental Disorders*, *50*(1), 189-198. https://doi.org/https://doi.org/10.1007/s10803-019-04245-0
51. Jo, C. W., Park, C. H., Lee, J. H., & Kim, J. H. (2017). Managing the behavior of a patient with autism by sedation via submucosal route during dental treatment. *J Dent Anesth Pain Med*, *17*(2), 157-161. https://doi.org/10.17245/jdapm.2017.17.2.157
52. Johnson, N. L., Bekhet, A., Robinson, K., & Rodriguez, D. (2014). Attributed meanings and strategies to prevent challenging behaviors of hospitalized children with autism: two perspectives. *J Pediatr Health Care*, *28*(5), 386-393. https://doi.org/10.1016/j.pedhc.2013.10.001
53. Johnson, N. L., & Bree, O. A. (2014). Social Script iPad Application Versus Usual Care Before Undergoing Medical Imaging: Two Case Studies of Children With Autism. *Journal of Radiology Nursing*, *33*(3), 121-126. https://doi.org/10.1016/j.jradnu.2014.04.001
54. Jung-Tang, N., Agrawal, R., Battersby, A., Burgoyne, L., Louise, J., & Cheung, A. (2023). Review of outpatient procedural sedation clinics in a tertiary paediatric hospital in South Australia. *J Paediatr Child Health*, *59*(10), 1160-1168. https://doi.org/10.1111/jpc.16478
55. Kaku, S. M., Bansal, S., Gs Rao, U., Bharath, R. D., Srinath, S., & Girimaji, S. C. (2023). Higher anesthetic dose requirement for sedation in children with autism spectrum disorder compared to neuro-atypical controls- a prospective observational study. *Research in Autism Spectrum Disorders*, *101*. https://doi.org/10.1016/j.rasd.2022.102086
56. Kamat, P. P., Bryan, L. N., McCracken, C. E., Simon, H. K., Berkenbosch, J. W., & Grunwell, J. R. (2018). Procedural sedation in children with autism spectrum disorders: A survey of current practice patterns of the society for pediatric sedation members. *Paediatr Anaesth*, *28*(6), 552-557. https://doi.org/10.1111/pan.13387
57. Kamat, P. P., Karaga, M. K., Wisniewski, B. L., McCracken, C. E., Simon, H. K., Sidhu, R., & Grunwell, J. R. (2018). Outpatient Procedural Sedation of Patients With Autism Spectrum Disorders for Magnetic Resonance Imaging of the Brain Using Propofol. *J Child Neurol*, *33*(5), 313-319. https://doi.org/10.1177/0883073817753908
58. Keidan, I., Ben-Menachem, E., Tzadok, M., Ben-Zeev, B., & Berkenstadt, H. (2015). Electroencephalography for children with autistic spectrum disorder: a sedation protocol. *Paediatr Anaesth*, *25*(2), 200-205. https://doi.org/10.1111/pan.12510
59. Kenneally, A., Cummins, M., Bailey, A., Yackey, K., Jones, L., Carter, C., Dugan, A., & Baum, R. A. (2023). Intranasal Dexmedetomidine Use in Pediatric Patients for Anxiolysis in the Emergency Department. *Pediatr Emerg Care*, *39*(9), 685-691. https://doi.org/10.1097/pec.0000000000002901
60. Kim, G., Carrico, C., Ivey, C., & Wunsch, P. B. (2019). Impact of sensory adapted dental environment on children with developmental disabilities. *Spec Care Dentist*, *39*(2), 180-187. https://doi.org/10.1111/scd.12360
61. Kim, T., Martinez, K., Cruz, B. L., Huang, J. S., & Stadnick, N. A. (2023). Caregiver Insights and Improvement Strategies for Youth with Autism Undergoing Gastrointestinal Endoscopy. *J Autism Dev Disord*, *53*(4), 1476-1482. https://doi.org/10.1007/s10803-021-05346-5
62. Kitt, E., Friderici, J., Kleppel, R., & Canarie, M. (2015). Procedural sedation for MRI in children with ADHD. *Paediatr Anaesth*, *25*(10), 1026-1032. https://doi.org/10.1111/pan.12721
63. Kittur, S., Basappa, N., Raju, O. S., Naik, S. V., & Shagale, A. M. (2022). Enhancing special care dentistry with sensory-adapted dental environment: A comparative study. *J Indian Soc Pedod Prev Dent*, *40*(3), 246-252. https://doi.org/10.4103/jisppd.jisppd_199_22
64. Klinepeter, E. A., Choate, J. D., Nelson Hall, T., & Gibbs, K. D. (2024). A "Whole Child Approach": Parent Experiences with Acute Care Hospitalizations for Children with Autism Spectrum Disorder and Behavioral Health Needs. *J Autism Dev Disord*. https://doi.org/10.1007/s10803-024-06573-2
65. Kurt, N., Caliskan, D. K., & Gunes, H. Y. (2022). Comparative Sedation with Sevoflurane and Thiopental in Children Undergoing MR Imaging. *J Coll Physicians Surg Pak*, *32*(11), 1381-1385. https://doi.org/10.29271/jcpsp.2022.11.1381
66. Kuschner, E. S., Kim, M., Bloy, L., Dipiero, M., Edgar, J. C., & Roberts, T. P. L. (2021). MEG-PLAN: a clinical and technical protocol for obtaining magnetoencephalography data in minimally verbal or nonverbal children who have autism spectrum disorder. *J Neurodev Disord*, *13*(1), 8. https://doi.org/10.1186/s11689-020-09350-1
67. Lewis, P., Wilson, N. J., Jaques, H., O’Reilly, K., & Wiese, M. (2019). A qualitative study of nurses’ perspectives of caring for children with intellectual disability and their families in a paediatric acute care setting. *Journal of Child Health Care*, *23*(4), 639-651. https://doi.org/10.1177/1367493519867234
68. Li, B. L., Yuen, V. M., Zhang, N., Zhang, H. H., Huang, J. X., Yang, S. Y., Miller, J. W., & Song, X. R. (2019). A Comparison of Intranasal Dexmedetomidine and Dexmedetomidine Plus Buccal Midazolam for Non-painful Procedural Sedation in Children with Autism. *J Autism Dev Disord*, *49*(9), 3798-3806. https://doi.org/10.1007/s10803-019-04095-w
69. Liddle, M., & Sonnentag, T. L. (2021). Effectiveness of Adaptive Care Plans for Children with Developmental Disabilities During Outpatient Clinic Appointments. *J Autism Dev Disord*, *51*(9), 3028-3038. https://doi.org/10.1007/s10803-020-04764-1
70. Luque, C. G., Atkins-Labelle, C., Pauwels, J., Costello, R., Kozak, F. K., & Chadha, N. K. (2021). Intranasal Dexmedetomidine increases the successful sedation of children with autism for out-patient auditory brainstem response hearing tests. *International Journal of Pediatric Otorhinolaryngology*, *151*, 110945. https://doi.org/https://doi.org/10.1016/j.ijporl.2021.110945
71. Mah, J. W., & Tsang, P. (2016). Visual Schedule System in Dental Care for Patients with Autism: A Pilot Study. *J Clin Pediatr Dent*, *40*(5), 393-399. https://doi.org/10.17796/1053-4628-40.5.393
72. Makhijani, A., Jenkins, N., Kaufman, J., Hoq, M., Priestley, S., Elia, S., McKenzie, I., Davidson, A., Leong, P., Lazzaro, T., McNab, S., & Danchin, M. (2024). Virtual reality for routine immunisations in needle phobic children with and without developmental disabilities: A pilot study. *Vaccine*, *42*(26), 126481. https://doi.org/10.1016/j.vaccine.2024.126481
73. Mangione, F., Bdeoui, F., Monnier-Da Costa, A., & Dursun, E. (2020). Autistic patients: a retrospective study on their dental needs and the behavioural approach. *Clin Oral Investig*, *24*(5), 1677-1685. https://doi.org/10.1007/s00784-019-03023-7
74. Marion, I. W., Nelson, T. M., Sheller, B., McKinney, C. M., & Scott, J. M. (2016). Dental stories for children with autism. *Spec Care Dentist*, *36*(4), 181-186. https://doi.org/10.1111/scd.12167
75. Martínez Pérez, E., Adanero Velasco, A., Gómez Clemente, V., Miegimolle Herrero, M., & Planells Del Pozo, P. (2023). Importance of Desensitization for Autistic Children in Dental Practice. *Children (Basel)*, *10*(5). https://doi.org/10.3390/children10050796
76. McBride, G. R., Stephenson, K. A. J., Comer, G., & Flanagan, O. (2021). The Use of Oral Midazolam to Facilitate the Ophthalmic Examination of Children with Autism and Developmental Disorders. *J Autism Dev Disord*, *51*(5), 1678-1682. https://doi.org/10.1007/s10803-020-04658-2
77. McConnell, K. L., Sassi, J. L., Carr, L., Szalwinski, J., Courtemanche, A., Njie‐Jallow, F., & Cheney, W. R. (2020). Functional analysis and generalized treatment of disruptive behavior during dental exams. *Journal of applied behavior analysis*, *53*(4), 2233-2249. https://doi.org/10.1002/jaba.747
78. McDonald, M., Gallaugher, S., Kammerer, E., & Ali, S. (2024). Creating a Low-Stimulus Clinic to improve immunization success rates for children with alternate environment needs: A quality improvement initiative. *Paediatr Child Health*, *29*(1), 17-22. https://doi.org/10.1093/pch/pxad081
79. Mehrotra, D., Shetty, A. A., Rai, K., & Kumara. (2024). Effect of audio and virtual reality distraction on the dental anxiety of children with mild intellectual disability. *Spec Care Dentist*, *44*(3), 868-877. https://doi.org/10.1111/scd.12932
80. Mellado-Cairet, P., Brebion, M., Henry, R., Crowe, A. M., Orliaguet, G., & Keita, H. (2024). Behavioral training and mirroring techniques to prepare for elective anesthesia in children with autism spectrum disorder: a prospective evaluation of implemented program. *Minerva Anestesiol*, *90*(9), 769-774. https://doi.org/10.23736/s0375-9393.24.18021-2
81. Miao, Y., Zheng, M., Li, Q., Xiong, L., Feng, J., Liu, X., Fan, G., Chaturvedi, R., Zhang, F., & Yin, N. (2024). Comparison of propofol‐esketamine versus propofol‐sufentanil for deep sedation and analgesia in children with autism: A randomized double‐blind clinical trial. *Autism Research*, *17*(7), 1356-1364. https://doi.org/10.1002/aur.3172
82. Morais, I., Rodrigues, J. T., Peixoto, C., Sousa, I., Mesquita, E., & Morais, A. (2019). Ambulatory Dental Procedures in Children with Intellectual Disability: A Ten-Year Review. *Ambulatory Surgery*, *25*(4), 110-113. https://search.ebscohost.com/login.aspx?direct=true&AuthType=ip,athens&db=ccm&AN=141211951&site=ehost-live
83. Morrissette, M., & Boman, J. (2020). Assessment of Aggressive Behaviour in a Patient with Autism Spectrum Disorder Requiring General Anesthesia. *J Can Acad Child Adolesc Psychiatry*, *29*(2), 106-109. https://pmc.ncbi.nlm.nih.gov/articles/PMC7213913/pdf/ccap29_p0106.pdf
84. Murdoch, L., & Chang, Y.-S. (2022). Parental experiences of caring for children who have learning disabilities and procedural anxiety in hospital: An interpretive phenomenological study. *Child: care, health and development*, *48*(5), 809-819. https://doi.org/10.1111/cch.12990
85. Murshid, E. Z. (2017). Effectiveness of a preparatory aid in facilitating oral assessment in a group of Saudi children with autism spectrum disorders in Central Saudi Arabia. *Saudi Med J*, *38*(5), 533-540. https://doi.org/10.15537/smj.2017.5.17398
86. Muskat, B., Burnham Riosa, P., Nicholas, D. B., Roberts, W., Stoddart, K. P., & Zwaigenbaum, L. (2014). Autism comes to the hospital: The experiences of patients with autism spectrum disorder, their parents and health-care providers at two Canadian paediatric hospitals. *Autism*, *19*(4), 482-490. https://doi.org/10.1177/1362361314531341
87. Muskat, B., Greenblatt, A., Nicholas, D. B., Ratnapalan, S., Cohen-Silver, J., Newton, A. S., Craig, W. R., Kilmer, C., & Zwaigenbaum, L. (2016). Parent and health care provider perspectives related to disclosure of autism spectrum disorder in pediatric emergency departments. *Autism*, *20*(8), 986-994. https://doi.org/10.1177/1362361315621520
88. Narzisi, A., Bondioli, M., Pardossi, F., Billeci, L., Buzzi, M. C., Buzzi, M., Pinzino, M., Senette, C., Semucci, V., Tonacci, A., Uscidda, F., Vagelli, B., Giuca, M. R., & Pelagatti, S. (2020). "Mom Let's Go to the Dentist!" Preliminary Feasibility of a Tailored Dental Intervention for Children with Autism Spectrum Disorder in the Italian Public Health Service. *Brain Sci*, *10*(7). https://doi.org/10.3390/brainsci10070444
89. Nelson, T., Chim, A., Sheller, B. L., McKinney, C. M., & Scott, J. M. (2017). Predicting successful dental examinations for children with autism spectrum disorder in the context of a dental desensitization program. *J Am Dent Assoc*, *148*(7), 485-492. https://doi.org/10.1016/j.adaj.2017.03.015
90. Nguyen, N., Pan, Z., Smith, C., & Friedlander, J. A. (2024). Transnasal endoscopy ease score "TNEase score" to evaluate patient tolerance of unsedated transnasal endoscopy. *J Pediatr Gastroenterol Nutr*, *78*(2), 381-385. https://doi.org/10.1002/jpn3.12102
91. Nicholas, D. B., Zwaigenbaum, L., Muskat, B., Craig, W. R., Newton, A. S., Cohen-Silver, J., Sharon, R. F., Greenblatt, A., & Kilmer, C. (2016). Toward Practice Advancement in Emergency Care for Children With Autism Spectrum Disorder. *Pediatrics*, *137 Suppl 2*, S205-211. https://doi.org/10.1542/peds.2015-2851S
92. Nicholas, D. B., Zwaigenbaum, L., Muskat, B., Craig, W. R., Newton, A. S., Kilmer, C., Greenblatt, A., Roberts, W., & Cohen-Silver, J. (2016). Experiences of emergency department care from the perspective of families in which a child has autism spectrum disorder. *Soc Work Health Care*, *55*(6), 409-426. https://doi.org/10.1080/00981389.2016.1178679
93. Nilchian, F., Shakibaei, F., & Jarah, Z. T. (2017). Evaluation of Visual Pedagogy in Dental Check-ups and Preventive Practices Among 6-12-Year-Old Children with Autism. *J Autism Dev Disord*, *47*(3), 858-864. https://doi.org/10.1007/s10803-016-2998-8
94. Nilsson, S., Brunsson, I., Askljung, B., Påhlman, M., & Himmelmann, K. (2017). A rectally administered combination of midazolam and ketamine was easy, effective and feasible for procedural pain in children with cerebral palsy. *Acta Paediatr*, *106*(3), 458-462. https://doi.org/10.1111/apa.13710
95. Nordahl, C. W., Mello, M., Shen, A. M., Shen, M. D., Vismara, L. A., Li, D., Harrington, K., Tanase, C., Goodlin-Jones, B., Rogers, S., Abbeduto, L., & Amaral, D. G. (2016). Methods for acquiring MRI data in children with autism spectrum disorder and intellectual impairment without the use of sedation. *J Neurodev Disord*, *8*, 20. https://doi.org/10.1186/s11689-016-9154-9
96. O'Brien, E. M., Stricker, P. A., Harris, K. A., Liu, H., Griffis, H., & Muhly, W. T. (2024). Perioperative Management and Outcomes in Patients With Autism Spectrum Disorder: A Retrospective Cohort Study. *Anesth Analg*, *138*(2), 438-446. https://doi.org/10.1213/ane.0000000000006426
97. Omer, R., Mohamed, N., & Peck, C. (2024). Oral health practices and challenges facing parents of autistic children in the Western Cape (2021). *Pediatric Dental Journal*, *34*(2), 55-61. https://doi.org/10.1016/j.pdj.2024.03.001
98. Ong, N., Ahuja, H., de Lima, J., Tomsic, G., Garg, P., Silove, N., Henao Urrego, B., & Weatherall, A. (2024). Perioperative pathways for children with neurodevelopmental conditions and behaviours that challenge: An evaluation of parent experiences for service improvement. *J Perioper Pract*, 17504589241253487. https://doi.org/10.1177/17504589241253487
99. Ong, N., Brogan, D., Lucien, A., Wolman, S., Campbell, D., Deng, L., Koirala, A., Garg, P., & Sharma, K. (2024). The development and evaluation of a vaccination pathway for children with intellectual and developmental disability and needle fear. *Paediatr Neonatal Pain*, *6*(1), 1-9. https://doi.org/10.1002/pne2.12103
100. Orellana, L., Martínez-Sanchis, S., & Silvestre, F. (2014). Training Adults and Children with an Autism Spectrum Disorder to be Compliant with a Clinical Dental Assessment Using a TEACCH-Based Approach...Treatment and Education of Autistic and related Communication-handicapped Children. *Journal of Autism & Developmental Disorders*, *44*(4), 776-785. https://doi.org/10.1007/s10803-013-1930-8
101. Oulton, K., Gibson, F., Kenten, C., Russell, J., Carr, L., Hassiotis, A., Kelly, P., Kerry, S., Tuffrey-Wijne, I., Whiting, M., & Wray, J. (2024). Being a child with intellectual disabilities in hospital: The need for an individualised approach to care. *J Appl Res Intellect Disabil*, *37*(1), e13153. https://doi.org/10.1111/jar.13153
102. Oulton, K., Sell, D., & Gibson, F. (2020). Hospitalized children with intellectual disability: Parents as partners in their care. *J Appl Res Intellect Disabil*, *33*(5), 917-926. https://doi.org/10.1111/jar.12713
103. Özkan, A. S., Erdoğan, M. A., Şanlı, M., Kaçmaz, O., Durmuş, M., & Çolak, C. (2015). Retrospective Evaluation of Dental Treatment under General Anaesthesia. *Turk J Anaesthesiol Reanim*, *43*(5), 332-336. https://doi.org/10.5152/tjar.2015.82542
104. Paasch, V., Leibowitz, L., Accardo, J., & Slifer, K. (2016). Preparing children with autism spectrum disorders for overnight sleep studies: A case series. *Clinical Practice in Pediatric Psychology*, *4*(2), 153-163. https://doi.org/10.1037/cpp0000139
105. Page, A., Gayson, C., Vanes, N., Ashmore, P., & McDonnell, A. (2017). Determining the sensory needs of children with Hunter and Sanfilippo syndromes who need hospital treatment. *Learning Disability Practice*, *20*(2), 17-21. https://doi.org/10.7748/ldp.2017.e1790
106. Parry, J., & Shepherd, J. (2018). Understanding oral health challenges for children and young people with autistic spectrum conditions: views of families and the dental team. *Journal of Disability & Oral Health*, *19*(4), 170-174. https://search.ebscohost.com/login.aspx?direct=true&AuthType=ip,athens&db=ccm&AN=134073974&site=ehost-live
107. Pascolo, P., Peri, F., Montico, M., Funaro, M., Parrino, R., Vanadia, F., Rusalen, F., Vecchiato, L., Benini, F., Congedi, S., Barbi, E., & Cozzi, G. (2018). Needle-related pain and distress management during needle-related procedures in children with and without intellectual disability. *Eur J Pediatr*, *177*(12), 1753-1760. https://doi.org/10.1007/s00431-018-3237-4
108. Pasley, K., Krivchenia, K., Dell, M. L., McCoy, K. S., & Paul, G. R. (2023). Clinical management of pediatric patients with cystic fibrosis and autism spectrum disorder. *Pediatr Pulmonol*, *58*(4), 1160-1168. https://doi.org/10.1002/ppul.26311
109. Pavlov, A., Hodnett, J. M., Booth, C., Wigton, S., Bernstein, A., Lomas Mevers, J., & Scheithauer, M. (2024). COVID-19 Vaccination Clinic for Individuals With Autism Spectrum Disorder and Related Disorders: Feasibility and Acceptability. *J Am Psychiatr Nurses Assoc*, *30*(3), 716-721. https://doi.org/10.1177/10783903231172997
110. Pecci-Lloret, M. P., Guerrero-Gironés, J., López-González, B., Rodríguez-Lozano, F. J., Oñate-Cabrerizo, D., Oñate-Sánchez, R. E., & Pecci-Lloret, M. R. (2021). Dental treatments under general anesthesia on children with special health care needs enrolled in the Spanish dental care program. *Journal of Clinical Medicine*, *10*(2), 1-9. https://doi.org/10.3390/jcm10020182
111. Pettersson, E., Christensen, B. M., Berglund, I. G., & Huus, K. (2023). Healthcare professionals' experiences of situations during a procedure with a child with autism spectrum disorder in the high-technology environment. *Child Care Health Dev*, *49*(6), 1087-1095. https://doi.org/10.1111/cch.13119
112. Pettersson, E., Christensen, B. M., Berglund, I. G., & Huus, K. (2024). Identifying actions taken by health care professionals during procedures involving children with autism spectrum disorders in a high technological environment: Using critical incident technique. *J Spec Pediatr Nurs*, *29*(4), e12438. https://doi.org/10.1111/jspn.12438
113. Ponde, V., Desai, A., Ekambaram, K., & Thakur, S. (2020). Dental procedure under opioid-free balanced anaesthesia in a child with Rett syndrome who convulsed on every attempt to feed: Case report. *Indian J Anaesth*, *64*(4), 325-327. https://doi.org/10.4103/ija.IJA_812_19
114. Prakash, S., Pai, V. K., Dhar, M., & Kumar, A. A. (2016). Premedication in an autistic, combative child: Challenges and nuances. *Saudi J Anaesth*, *10*(3), 339-341. https://doi.org/10.4103/1658-354x.174917
115. Pua, E. P. K., Barton, S., Williams, K., Craig, J. M., & Seal, M. L. (2020). Individualised MRI training for paediatric neuroimaging: A child-focused approach. *Dev Cogn Neurosci*, *41*, 100750. https://doi.org/10.1016/j.dcn.2019.100750
116. Sahyoun, C., Krauss, B., Bevacqua, M., Antonsen, A., Jardinier, L., & Barbi, E. (2023). Safety and Efficacy Associated With a Family-Centered Procedural Sedation Protocol for Children With Autism Spectrum Disorder or Developmental Delay. *JAMA Netw Open*, *6*(5), e2315974. https://doi.org/10.1001/jamanetworkopen.2023.15974
117. Salam, T. A. A., Ummer, M., Abdullah Alowairdhi, A., Khalid Alsubait, A., Marwan Aljuhani, S., Abdullah Alzahrani, A., & Ali Alqahtani, A. (2023). Management of Attention-Deficit Hyperactivity Disorder Children for Dental Procedures. *Cureus*, *15*(4), e36989. https://doi.org/10.7759/cureus.36989
118. Sarapultseva, M., & Sarapultsev, A. (2024). Comprehensive dental care in patient with Rubinstein-Taybi syndrome: A 3-year case study using progressive desensitization techniques and oral sedation. *Spec Care Dentist*, *44*(6), 1589-1599. https://doi.org/10.1111/scd.13050
119. Sathyamoorthy, M., Hamilton, T. B., Wilson, G., Talluri, R., Fawad, L., Adamiak, B., Wallace, C., Borissova, I., & Heard, C. (2019). Pre-medication before dental procedures: A randomized controlled study comparing intranasal dexmedetomidine with oral midazolam. *Acta Anaesthesiol Scand*, *63*(9), 1162-1168. https://doi.org/10.1111/aas.13425
120. Sawicki, C. M., Pielech, M., & Wade, S. D. (2023). Practice Patterns Among Dentist Anesthesiologists for Pediatric Patients with Autism Spectrum Disorders. *Pediatr Dent*, *45*(1), 37-53. https://pmc.ncbi.nlm.nih.gov/articles/PMC10262783/pdf/nihms-1903674.pdf
121. Schreiber, S., Cozzi, G., Rutigliano, R., Assandro, P., Tubaro, M., Cortellazzo Wiel, L., Ronfani, L., & Barbi, E. (2016). Analgesia by cooling vibration during venipuncture in children with cognitive impairment. *Acta Paediatrica, International Journal of Paediatrics*, *105*(1), e12-e16. https://doi.org/10.1111/apa.13224
122. Seo, K. H., Jung, H. S., Kang, E. G., Kim, C. J., Rhee, H. Y., & Jeon, Y. S. (2014). Sedation using 5% lidocaine patches, midazolam and propofol in a combative, obese adolescent with severe autistic disorder undergoing brain magnetic resonance imaging: a case report. *Korean J Anesthesiol*, *67*(6), 421-424. https://doi.org/10.4097/kjae.2014.67.6.421
123. Sfriso, F., Bonardi, C. M., Viaggi, F., Sartori, S., Boniver, C., Martinolli, F., Da Dalt, L., Frigo, A. C., Mazza, A., & Amigoni, A. (2020). Dexmedetomidine for EEG sedation in children with behavioral disorders. *Acta Neurol Scand*, *142*(5), 493-500. https://doi.org/10.1111/ane.13293
124. Shokri, H., & Kasem, A. A. (2019). Dexmedetomidine versus midazolam sedation for autistic children undergoing electroencephalogram: a prospective randomized trial. *AIN SHAMS JOURNAL OF ANESTHESIOLOGY*, *11*(1). https://doi.org/10.1186/s42077-019-0047-5
125. Snow, S. L., Smith, I. M., Latimer, M., Stirling Cameron, E., Fox, J., & Chorney, J. (2022). A balancing act: An interpretive description of healthcare providers' and families' perspective on the surgical experiences of children with autism spectrum disorder. *Autism*, *26*(4), 839-848. https://doi.org/10.1177/13623613211034057
126. Stein Duker, L. I., Como, D. H., Jolette, C., Vigen, C., Gong, C. L., Williams, M. E., Polido, J. C., Floríndez-Cox, L. I., & Cermak, S. A. (2023). Sensory Adaptations to Improve Physiological and Behavioral Distress During Dental Visits in Autistic Children: A Randomized Crossover Trial. *JAMA Netw Open*, *6*(6), e2316346. https://doi.org/10.1001/jamanetworkopen.2023.16346
127. Swartz, J. S., Amos, K. E., Brindas, M., Girling, L. G., & Ruth Graham, M. (2017). Benefits of an individualized perioperative plan for children with autism spectrum disorder. *Paediatr Anaesth*, *27*(8), 856-862. https://doi.org/10.1111/pan.13189
128. Swiggum, M., & Grant, L. (2019). Monitoring Procedural Pain and Distress in a Child With Rett Syndrome: A Case Report. *Pediatr Phys Ther*, *31*(4), E1-e5. https://doi.org/10.1097/pep.0000000000000651
129. Taghizadeh, N., Heard, G., Davidson, A., Williams, K., & Story, D. (2019). The experiences of children with autism spectrum disorder, their caregivers and health care providers during day procedure: A mixed methods study. *Paediatr Anaesth*, *29*(9), 927-937. https://doi.org/10.1111/pan.13689
130. Talley, M., Brown, C., Wingo, N., Conway, J., Maha, J., & Kong, M. (2024). Neurodivergent patient experience in a tertiary children's hospital-a qualitative analysis. *Front Pediatr*, *12*, 1427433. https://doi.org/10.3389/fped.2024.1427433
131. Tetef, S. (2014). Effectiveness of Transmucosal Sedation for Special Needs Populations in the Ambulatory Care Setting. *AORN Journal*, *100*(6), 651-669. https://doi.org/10.1016/j.aorn.2014.04.019
132. Thomas, N., Blake, S., Morris, C., & Moles, D. R. (2018). Autism and primary care dentistry: parents' experiences of taking children with autism or working diagnosis of autism for dental examinations. *International journal of paediatric dentistry*, *28*(2), 226-238. https://doi.org/10.1111/ipd.12345
133. Thompson, D. G., & Tielsch-Goddard, A. (2014). Improving management of patients with autism spectrum disorder having scheduled surgery: optimizing practice. *J Pediatr Health Care*, *28*(5), 394-403. https://doi.org/10.1016/j.pedhc.2013.09.007
134. Thunberg, G., Buchholz, M., & Nilsson, S. (2016). Strategies that assist children with communicative disability during hospital stay. *Journal of Child Health Care*, *20*(2), 224-233. https://doi.org/10.1177/1367493514568298
135. Thunberg, G., Törnhage, C. J., & Nilsson, S. (2016). Evaluating the Impact of AAC Interventions in Reducing Hospitalization-related Stress: Challenges and Possibilities. *Augment Altern Commun*, *32*(2), 143-150. https://doi.org/10.3109/07434618.2016.1157703
136. Tounsi, A. (2017). CHILDREN WITH AUTISM SPECTRUM DISORDERS CAN BE SUCCESSFULLY EXAMINED USING DENTAL DESENSITIZATION. *JOURNAL OF EVIDENCE-BASED DENTAL PRACTICE*, *17*(4), 414-415. https://doi.org/10.1016/j.jebdp.2017.10.007
137. Tziraki, M., Garg, S., Harrison, E., Wright, N. B., Hawkes, R., Akhtar, K., Green, J., & Stivaros, S. (2021). A Neuroimaging Preparation Protocol Tailored for Autism. *Autism Res*, *14*(1), 65-74. https://doi.org/10.1002/aur.2427
138. Wasfy, S. F., Hassan, R. M., & Hashim, R. M. (2020). Effectiveness and safety of Ketamine and Midazolam mixture for procedural sedation in children with mental disabilities: A randomized study of intranasal versus intramuscular route. *Egyptian Journal of Anaesthesia*, *36*(1), 16-23. https://doi.org/10.1080/11101849.2020.1727669
139. Whippey, A., Bernstein, L. M., O'Rourke, D., & Reddy, D. (2019). Enhanced perioperative management of children with autism: a pilot study. *Can J Anaesth*, *66*(10), 1184-1193. https://doi.org/10.1007/s12630-019-01410-y
140. Winterberg, A. V., Jones, E., Ding, L. L., Hill, L. M., & Varughese, A. M. (2022). Adaptive Care for Perioperative Patients With Developmental Disabilities: An Exploration of Interventions and Family. *Journal of Pediatric Health Care*, *36*(6), 529-539. https://doi.org/10.1016/j.pedhc.2022.05.019
141. Wittling, K., Dufur, J. P., McClain, A., & Gettis, M. (2018). Behavioral Coping Plans: One Inter-Professional Team's Approach to Patient-Centered Care. *J Pediatr Nurs*, *41*, 135-139. https://doi.org/10.1016/j.pedn.2018.05.004
142. Wood, E. B., Halverson, A., Harrison, G., & Rosenkranz, A. (2019). Creating a Sensory-Friendly Pediatric Emergency Department. *J Emerg Nurs*, *45*(4), 415-424. https://doi.org/10.1016/j.jen.2018.12.002
143. Yamada, K., Suzuki, Y., Ueki, S., Itoh, K., Watanabe, M., Suzuki, K., & Igarashi, H. (2020). Participant-driven Simulation Protocol With a Mock Scanner for Pediatric Magnetic Resonance Neuroimaging Preparation Without Sedation. *Clinical Simulation in Nursing*, *47*, 40-47. https://doi.org/10.1016/j.ecns.2020.07.002
144. Zink, A. G., Molina, E. C., Diniz, M. B., Santos, M., & Guaré, R. O. (2018). Communication Application for Use During the First Dental Visit for Children and Adolescents with Autism Spectrum Disorders. *Pediatr Dent*, *40*(1), 18-22.

**Supplementary Material: Study Description of Included Studies**

**Supplementary Table 2. Frequency of Procedural Support Strategies and Their Use Across Clinical Specialties**

| Types of Procedural Support |  | Specialities |
| --- | --- | --- |
| Visit Preparation and Support | 61 (42.4) | Dental (19); Medical/Surgical (15); Emergency Department (2); Perioperative (9); Radiology (7); Anaesthesiology (1); Psychiatry (3); Other (5) |
| Pharmacological Agents | 49 (34.0) | Dental (15); Medical/Surgical (13); Emergency Department (4); Perioperative (6); Radiology (4); Neurology (3); Anaesthesiology (1); Other (4) |
| Patient Care Plan or Pathway | 43 (29.9) | Dental (2); Medical/Surgical (19); Emergency Department (2); Perioperative (11); Radiology (4); Anaesthesiology (1); Psychiatry (1); Other (6) |
| Hospital-wide Strategy | 19 (13.2) | Dental (1); Medical/Surgical (4); Emergency Department (5); Perioperative (4); Radiology (2); Anaesthesiology (1); Other (1) |
| Behavioural Intervention | 18 (12.5) | Dental (13); Medical/Surgical (1); Psychiatry (2); Other (2) |
| Technology (VR) | 16 (11.1) | Dental (8); Medical/Surgical (2); Perioperative (1); Radiology (2); Psychiatry (1); Other (1) |
| Environmental Modification (i.e low stimulus environment) | 16 (11.1) | Dental (5); Medical/Surgical (5); Emergency Department (1); Perioperative (3); Other (1) |
| Distraction (i.e audio-visual) | 13 (9.0) | Dental (3); Medical/Surgical (6); Physical Rehabilitation (2); Emergency Department (1); Neurology (1) |
| Play-based | 9 (6.3) | Dental (2); Medical/Surgical (2); Perioperative (1); Radiology (2); Neurology (1); Psychiatry (1) |
| Communication Support | 4 (2.8) | Medical/Surgical (2); Other (1); Psychiatry (1) |
| Staff Training/Education | 4 (2.8) | Dental (1); Medical/Surgical (2); Emergency Department (1) |
| Specialist Support | 4 (2.8) | Medical/Surgical (2); Dental (1); Emergency Department (1) |

Note: Percentages are calculated based on the 144 total interventions identified across all studies. Some studies implemented multiple support strategies, so the sum of percentages exceeds 100%.

**Supplementary Table 3. Support Strategies by Provider Type for Children with Neurodevelopmental Conditions**

| Support Strategy | n (%) | Provider Types |
| --- | --- | --- |
| Visit Preparation and Support | 61 (42.4) | Parent/Caregiver (n=41); Nurse (n=30); Medical Doctor (n=13); Dentist (n=10); Other Specialist (n=9); Child Life Specialist (n=7); Anaesthesiologist (n=4); Radiologist (n=3) |
| Pharmacological Agents | 49 (34.0) | Medical Doctor (n=31); Nurse (n=18); Dentist (n=17); Anaesthesiologist (n=12); Parent/Caregiver (n=5); Physiotherapist (n=2); Radiologist (n=1) |
| Patient Care Plan or Pathway | 43 (29.9) | Medical Doctor (n=26); Parent/Caregiver (n=24); Nurse (n=18); Child Life Specialist (n=10); Multidisciplinary Team (n=8); Anaesthesiologist (n=7); Other Specialist (n=7); Radiologist (n=2) |
| Hospital-wide Strategy | 19 (13.2) | Medical Doctor (n=11); Nurse (n=10); Multidisciplinary Team (n=7); Staff Training Professional (n=5); Parent/Caregiver (n=3) |
| Behavioural Intervention | 18 (12.5) | Parent/Caregiver (n=8); Dentist (n=5); Other Specialist (n=4); Child Life Specialist (n=2) |
| Technology (VR) | 16 (11.1) | Dentist (n=4); Medical Doctor (n=3); Other Specialist (n=3); Parent/Caregiver (n=3); Nurse (n=2); Radiologist (n=1) |
| Environmental Modification | 16 (11.1) | Parent/Caregiver (n=6); Nurse (n=4); Child Life Specialist (n=4); Medical Doctor (n=3); Dentist (n=3); Other Specialist (n=3) |
| Distraction (audio-visual) | 13 (9.0) | Nurse (n=5); Parent/Caregiver (n=5); Dentist (n=4); Other Specialist (n=3); Physiotherapist (n=1) |
| Play-based | 9 (6.3) | Other Specialist (n=4); Parent/Caregiver (n=3); Child Life Specialist (n=2) |
| Communication Support | 4 (2.8) | Nurse (n=2); Multidisciplinary Team (n=2); Parent/Caregiver (n=2); Staff Training Professional (n=1) |
| Staff Training/Education | 4 (2.8) | Staff Training Professional (n=4); Medical Doctor (n=2); Multidisciplinary Team (n=2) |
| Specialist Support | 4 (2.8) | Medical Doctor (n=2); Nurse (n=2); Child Life Specialist (n=2); Other Specialist (n=2) |

Note: Based on 144 studies. Many support strategies were implemented by multiple providers, so the sum across rows exceeds the total count for each strategy. Percentages indicate the proportion of studies implementing each support strategy.

**Supplementary Table 4. Pharmacological Agents by Procedure Type with Dosage Ranges**

| **Pharmacological Agent** | **Dosage Range** | **Route** | **Procedure Types (n)** |
| --- | --- | --- | --- |
| **Sedation** | Unspecified | Unspecified | Surgery (1); Dental (1); General Hospital Experience (1); Needle-related procedures (1); Radiological/diagnostic (1) |
| **Non-prescription Medication** | Unspecified | Unspecified | Physical Rehabilitation (1) |
| **Anxiolytic or Analgesic Premedication** | Unspecified | Oral | Dental (4); Surgery (1) |
| **Dexmedetomidine** | 1-4 μg/kg | Intranasal | Radiological/Diagnostic (4); Dental (2); Emergency (1) Surgery (1) Needle-related procedures (3) |
|  | unspecified | Intranasal | Needle-related procedures (1), Radiological/Diagnostic (1) |
|  | 0.5-2 μg/kg | Intravenous | Radiological/Diagnostic (6) |
|  | Unspecified | Intravenous | Radiological/Diagnostic (1) |
|  | 4 μg/kg | Intramuscular | Emergency (1); Radiological/Diagnostic (1) |
| **Propofol** | 0.5-3 mg/kg bolus | Intravenous | Radiological/Diagnostic (6); Surgery (1) |
|  | unspecified | Intravenous | Dental (3); Radiological/Diagnostic (3); Needle-related procedures (1); Emergency (1); Surgery (1) |
|  | 5-6mg/kg/hr | Continuous Infusion | Radiological/Diagnostic (3) |
| **Midazolam** | 0.25-0.5 mg/kg (max 15 mg) | Oral | Dental (4); Radiological/Diagnostic (3); Needle-related procedures (2) Emergency (1) Surgery (1) |
|  | Unspecified Dose | Oral | Radiological/Diagnostic (2); Surgery (1); Needle-related procedures (1); Emergency (1) |
|  | 0.05-0.45 mg/kg | Intravenous | Emergency (1); Radiological/Diagnostic (6); Needle-related procedures (1) |
|  | unspecified | Intravenous | Dental (1) |
|  | 0.1-0.25mg/kg | Submucosal/Buccal | Dental (2); Radiological/Diagnostic (1) |
|  | 0.15-0.37 mg/kg | Rectal | Needle-related procedures (1) |
|  | 0.15-0.2mg/kg | Intramuscular | Dressing Changes (1); Dental (1), Radiological/Diagnostic (2) |
|  | unspecified | intramuscular | Radiological/Diagnostic (1); Emergency (1) |
|  | 0.2-0.5mg/kg | Intranasal/transmucosal | Dressing Changes (1); Dental (2), Radiological/Diagnostic (2); Needle-related procedures (2); Emergency (1); Surgery (1) |
|  | Unspecified dose | Intranasal | Radiological/Diagnostic (1); Needle-related procedures (1); Surgery (1) |
| **Ketamine** | 0.7-2.2 mg/kg | Intravenous | Needle-related procedures (1); Emergency (1); Radiological/Diagnostic (2) |
|  | unspecified | Intravenous | Radiology/Diagnostic (1) |
|  | 2.5-4.9 mg/kg (mean 3.9) | Rectal | Needle-related procedures (1) |
|  | 5-7mg/kg | Oral | Dental (1); Needle-related procedures (1); Surgery (1) |
|  | Not specified | Oral (mixed in juice) | Emergency (1); Radiological/Diagnostic (1) |
|  | 3.2-5mg/kg | Intramuscular | Radiological/Diagnostic (2); Dental (1); Dressing Change (1); Emergency (1) |
|  | unspecified | intramuscular | Radiological/Diagnostic (2); Dental (1); Emergency (1); Needle-related procedures (1) |
|  | 2-5mg/kg | Intranasal | Dental (2); Dressing Change (1); Radiological/Diagnostic (1); Needle-related procedures (1) |
| **Esketamine** | 0.3mg/kg | intravenous | Radiological/Diagnostic (1) |
| **General Anaesthesia** | Protocol-based | Inhalational | Dental (4); Surgery (2) |
|  | Protocol-based | Total IV | Dental (2); Surgery (2); Radiological/Diagnostic (5); Needle-related Procedures (1) |
|  | Protocol-based | Oral | Emergency (1); Radiological/Diagnostic (1) |
|  | Protocol-based | Unspecified | Dental (8) |
| **Thiopental** | 3mg/kg bolus | Intravenous | Radiological/Diagnostic (1) |
|  | Unspecified | Intravenous | Dental (1); Surgery (1); Radiological/Diagnostic (1) |
| **Lidocaine** | 0.5-1mg/kg (max 10mg) | Intravenous | Dental (1); Surgery (1); Radiological/Diagnostic (1) |
| **Sevoflurane** | Unspecified | Inhalation with face mask | Dental (3); Surgery (2); Radiological/Diagnostic (1) |
| **Chloral Hydrate** | 50-75mg/kg (maximum 1000mg) | Oral | Radiological/Diagnostic (2) |
| **Nitrous Oxide** | 50%-70% mixture with oxygen | Inhalational | Dental (5); General Hospital Experience (1); Physical Rehabilitation (1); Needle Related Procedures (4); Radiological/Diagnostic (3) |
| **Clonidine** | Unspecified | Oral | Surgery (1); Needle-related procedure (1); Dental (1) |
| **Other benzodiazepines** | Lorazepam, dose unspecified | Intramuscular | Emergency (1); Radiological/Diagnostic (1) |
|  | Temazepam  Unspecified Dose | Oral | Surgery (1) |
|  | Diazepam 5-10mg | Oral | Needle-related procedure (1) |
|  | Clonazepam 2.5mg | Oral | Needle-related procedure (1) |
| **Sedating Antihistamine** | Phenergan, 2.5mg | Oral | Needle-related procedure (1) |
|  | Promethazine, Dose unspecified | Unspecified | Dental (1) |
| **Local Anaesthetic** | 2-5% Lidocaine | Topical (cream or spray) | Radiology/Diagnostic (2); Needle-related procedures (3); Dental (2); Surgery (1) |
|  | Unspecified | Injection/infiltration | Dental (3); Surgical (1) |
| **Antipsychotic** | Haloperidol Unspecified dose | Intramuscular | Radiological/Diagnostic (2) |
|  | Olanzapine 5-10mg | Oral | Needle-related procedure (1) |
|  | Olanzapine, dose unspecified | Oral | Emergency (1); Radiological/Diagnostic (1) |
|  | Risperidone, 2.5mg | Oral | Needle-related procedure (1) |
| **Opioid** | Morphine, unspecified dose | Unspecified | Dental (1); Surgery (1) |
|  | Tramadol, dose unspecified | unspecified | Dental (1); Surgery (1) |
|  | Fentanyl, dose unspecified | unspecified | Needle-related procedure (1); Emergency (1); Radiological/Diagnostic (3); Dental (1) |
|  | Pethidine, unspecified dose | unspecified | Dental (1) |
|  | Sufentanil 0.2mcg/kg | Intravenous | Radiological/Diagnostic (1) |

**Supplementary Table 5. Adverse Events Associated with Procedural Support Strategies**

| Support Strategy | Studies Reporting Adverse Events (n) | Examples of Adverse Events Reported |
| --- | --- | --- |
| Pharmacological Agents | 26 | Cardiovascular effects: Hypotension; Bradycardia |
|  |  | Respiratory effects: Respiratory depression; Increased secretions; Oxygen desaturation |
|  |  | Emergence phenomena: Agitation/delirium; Nightmares |
|  |  | Gastrointestinal effects: Vomiting; Nausea |
|  |  | Medication-specific effects: Injection pain; Rash; Limb tremors; Headache |
|  |  | Procedural complications: Inadequate sedation; Oversedation/prolonged sedation; Difficult intubation |
| Visit Preparation and Support | 6 | Behavioural/emotional responses: Anxiety during preparation; Distress requiring rescheduling; Emotional meltdowns post-procedure |
|  |  | Practical challenges: Long wait times increasing distress; Prolonged preparation times |
| Technology (VR) | 1 | User experience issues: Sense of loss of control; Anxiety from vision obstruction; Discontinuation of VR use |
| Environmental Modification | 0 | None reported |
| Distraction Techniques | 1 | Device-related issues: Difficult access from vibration; Arm stiffness from cold |
| Physical Restraint | 3 | Patient distress: Resistance and behavioural escalation; Need for additional sedation |
| Behavioural Intervention | 1 | Avoidance behaviours: Refusal to participate |
| Hospital-wide Strategy | 1 | Implementation challenges: Parent concerns about accommodations; Staff training limitations |
| Patient Care Plan or Pathway | 1 | Care coordination issues: Increased sensory sensitivity in care areas; Caregiver distress from poor explanation |

Note: Frequencies are provided when explicitly reported in studies; NR indicates not reported quantitatively. Multiple studies reported multiple types of adverse events. The most common adverse events were associated with pharmacological interventions, while non-pharmacological interventions showed fewer and generally less severe adverse events.
